# Human intraepithelial mast cell differentiation and effector function are directed by TGF-**β** signaling

**DOI:** 10.1172/JCI174981

**Published:** 2025-01-02

**Authors:** Tahereh Derakhshan, Eleanor Hollers, Alex Perniss, Tessa Ryan, Alanna McGill, Jonathan Hacker, Regan W. Bergmark, Neil Bhattacharyya, Stella E. Lee, Alice Z. Maxfield, Rachel E. Roditi, Lora Bankova, Kathleen M. Buchheit, Tanya M. Laidlaw, Joshua A. Boyce, Daniel F. Dwyer

**Affiliations:** 1 Jeff and Penny Vinik Center for Allergic Disease Research, Division of Allergy and Clinical Immunology, Brigham and Women’s Hospital, Boston, Massachusetts, USA.; 2 Harvard Medical School, Boston, Massachusetts, USA.; 3 Department of Surgery, Brigham and Women’s Hospital, Boston, Massachusetts, USA.; 4 Department of Otolaryngology, Massachusetts Eye and Ear, Boston, Massachusetts, USA.

**Keywords:** Immunology, Allergy, Asthma, Mast cells

## Abstract

Mast cells (MCs) expressing a distinctive protease phenotype (MC_T_s) selectively expand within the epithelium of human mucosal tissues during type 2 (T2) inflammation. While MC_T_s are phenotypically distinct from subepithelial MCs (MC_TC_s), signals driving human MC_T_ differentiation and this subset’s contribution to inflammation remain unexplored. Here, we have identified TGF-β as a key driver of the MC_T_ transcriptome in nasal polyps. We found that short-term TGF-β signaling alters MC cell surface receptor expression and partially recapitulated the in vivo MC_T_ transcriptome, while TGF-β signaling during MC differentiation upregulated a larger number of MC_T_-associated transcripts. TGF-β inhibited the hallmark MC_TC_ proteases chymase and cathepsin G at both the transcript and protein level, allowing selective in vitro differentiation of MC_T_s for functional study. We identified discrete differences in effector phenotype between in vitro–derived MC_T_s and MC_TC_s, with MC_T_s exhibiting enhanced proinflammatory lipid mediator generation and a distinct cytokine, chemokine, and growth factor production profile in response to both innate and adaptive stimuli, recapitulating functional features of their tissue-associated counterpart MC subsets. Thus, our findings support a role for TGF-β in promoting human MC_T_ differentiation and identified a discrete contribution of this cell type to T2 inflammation.

## Abstract 2

Human intraepithelial MCs have a distinct protease, transcriptional, and effector phenotype that is driven by TGF-β signaling during mast cell differentiaiton

## Introduction

Mast cells (MCs) are tissue-resident granulocytes thought to play key roles in type 2 (T2) inflammatory diseases, including asthma and chronic rhinosinusitis (CRS). MCs originate from rare circulating progenitors that mature in peripheral tissue, where they acquire histochemically distinct phenotypes based on their tissue localization (1). Human MCs are classically categorized based on the protease content of their secretory granules. Subepithelial MCs (MC_TC_s), possessing tryptase, chymase, cathepsin G (CTSG), and carboxypeptidase A (CPA3), are found in the skin, the submucosa of the lung and digestive tissue, and proximal to vasculature and peripheral nerves. MCs possessing tryptase alone under homeostatic conditions (MC_T_s) constitute the main MC population in the airway and digestive epithelium and the pulmonary alveolar parenchyma (2). We recently reported that MC_T_s and MC_TC_s in CRS with nasal polyposis (CRSwNP) represent poles along a transcriptional gradient linked by a transitional intermediate, suggesting divergence from a common progenitor polarizing in response to microenvironmental signals (3). However, the identity of these polarizing signals and the role of each subset in tissue inflammation remain undefined. Further, whether MC_T_s and MC_TC_s are terminally differentiated or can switch phenotypic or functional characteristics in response to environmental changes is unknown.

Clinical studies suggest a central role for MC_T_s in driving T2 mucosal disease pathobiology. MC_T_s preferentially expand within the epithelium during T2 inflammation (4–7), where they further express CPA3 in T2-high asthma, CRSwNP, and eosinophilic esophagitis (EoE) (4, 5, 8). Intraepithelial MC burden correlates with airway reactivity to cold air- and exercise-induced bronchoconstriction in asthmatic patients, while elevated expression of MC-specific transcripts in the bronchial brushings of T2-high asthmatic patients (reflecting intraepithelial MC_T_s) predicts improved responsiveness to corticosteroid treatment, a therapy that decreases intraepithelial MC_T_ concentration but minimally impacts MC_TC_s (8–10). Thus, determining the factors that direct MC_T_ differentiation and how they participate in tissue inflammation has important therapeutic implications for selectively targeting their contribution to disease.

Previously, we identified a role for TGF-β in driving differentiation of murine mucosal MCs, which like human MC_T_s, expand during T2 pulmonary inflammation (11). However, the role of TGF-β in human MC development and the contribution of each MC phenotype to inflammation remained unexplored. Here, we find that MC_T_s in CRSwNP express TGF-β target transcripts, while TGF-β treatment elicits discrete, time-dependent transcriptional phenotypes in peripheral blood–derived (PB-derived) MCs (PB-MCs). TGF-β–driven transcriptional changes in vitro are highly reflective of the in vivo MC_T_ transcriptome, an effect that increases with exposure duration. These changes include both suppressing transcripts encoding MC_TC_-associated proteases (*CMA1*, *CTSG*) and enhancing transcripts encoding MC_T_-associated transcription factors (*SKIL*, *ELK3*, *HEY1*, *NFATC1*) and lipid mediator biosynthetic pathway components (*ALOX5AP*, *PTGS1*). Culturing PB CD34^+^ cells under MC-differentiating conditions in the presence of TGF-β1 led to the development of MC_T_-like cells with reduced intracellular chymase and CTSG. The protease phenotype of in vitro–derived MC_T_s was strikingly plastic, with TGF-β1 withdrawal leading to chymase induction, an effect also observed in sorted CRSwNP MC_T_s cultured ex vivo in the absence of TGF-β1. TGF-β1 elicited duration-dependent effects on MC effector function, inhibiting MRGPRX2 expression and function, altering profiles of cytokine and chemokine secretion following activation, and enhancing eicosanoid production, mirroring differences observed between MC_T_s and MC_TC_s in vivo. Thus, our findings indicate that human MC subsets have distinct capacity to shape disease pathogenesis through proinflammatory mediator production and identify a central role for TGF-β signaling in both directing MC_T_ differentiation and maintaining their phenotype.

## Results

### Defining human MC phenotypes in nasal polyposis.

**Figure 1:**
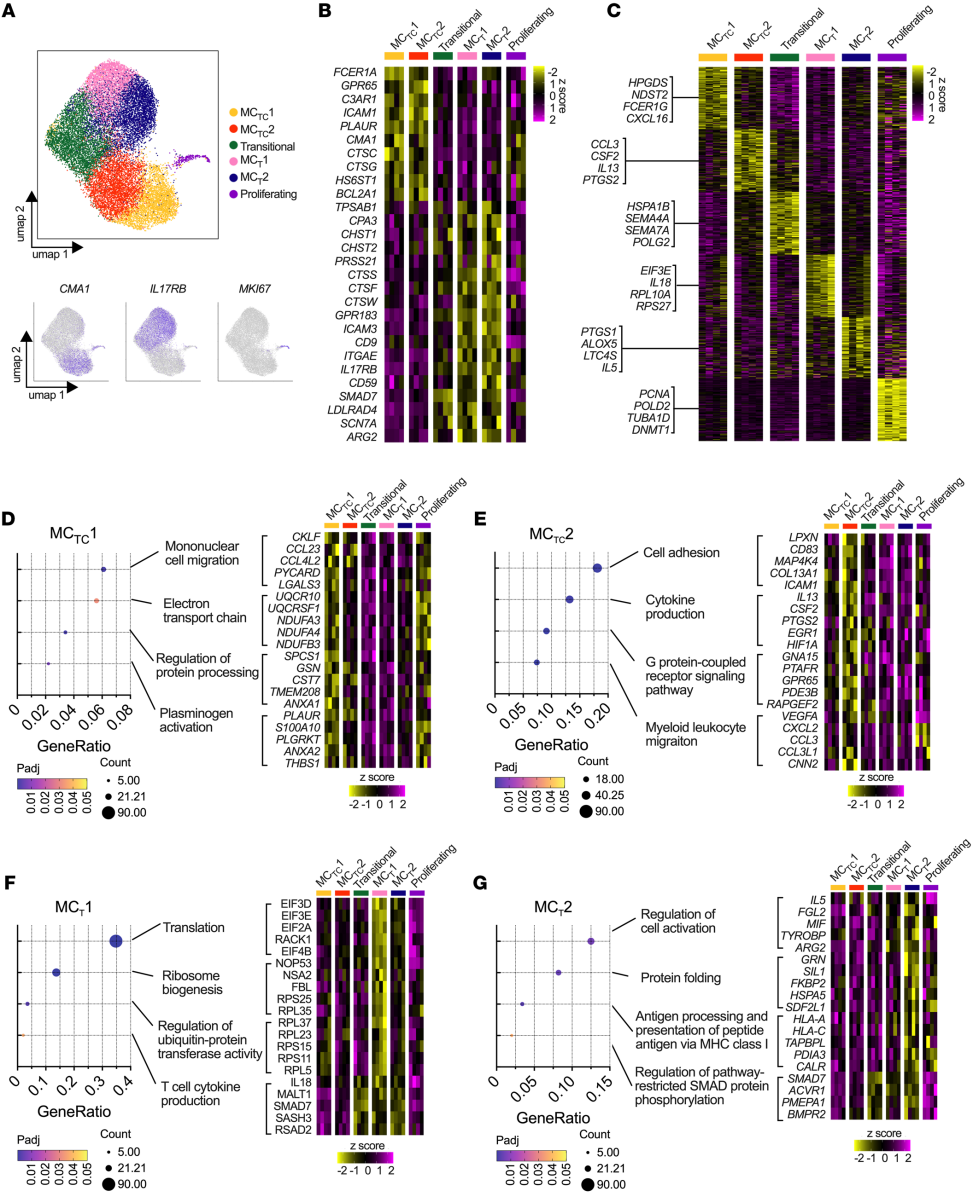
Phenotypic heterogeneity of MCs is marked by expression of distinct transcriptional cassettes. (**A**) Uniform manifold approximation and projection (UMAP) depiction of 6 MC clusters identified through scRNA-Seq analysis of MCs sorted from 4 AERD patients (top), with expression patterns for select subset-associated transcripts (bottom). (**B**) Row-normalized heatmap of common genes expressed by MC_TC_ and MC_T_ clusters (FDR < 0.05, log_2_FoldChange > 0.5, DESeq2) (**C**) Row-normalized heatmap of top differentially expressed genes across clusters (FDR < 0.05, log_2_FoldChange > 0.5, DESeq2), with representative cluster-enriched genes highlighted. (**D**–**G**) Enrichment of biological processes in (**D**) MC_TC_1, (**E**) MC_TC_2, and (**F**) MC_T_1, and (**G**) MC_T_2 clusters with row-normalized heatmaps showing expression of select process-associated genes. Heatmap columns indicate average cluster expression for each of *n* = 4 individuals; scale bars denote *z* score.

To gain deeper insights into the developmental signals directing MC differentiation and heterogeneity in severe T2 inflammation, MCs were flow sorted from the nasal polyps of 4 patients with aspirin exacerbated respiratory disease (AERD), a disease associated with refractory eosinophilic nasal polyposis and asthma. MCs were sorted based on canonical surface markers (CD45^+^, CD117^+^, FcɛR1α^+^, lineage [CD11b^–^, CD11c^–^, CD3^–^, CD19^–^]) for single-cell RNA-Seq (scRNA-Seq) using the 10x Genomics, version 3, chromium platform (Supplemental Figure 1A; supplemental material available online with this article; https://doi.org/10.1172/JCI174981DS1), taking advantage of the increased transcript detection and recovery available with the platform chemistry relative to earlier platforms. MCs were identified through protease transcript expression (*TPSAB1*, *CPA3*), computationally separated from contaminating populations and a small donor-restricted cluster enriched for interferon signature genes (Supplemental Figure 1, B and C), and integrated using the Harmony software package, version 1.2 (12). We identified 6 MC clusters, including 2 MC_TC_ and 2 MC_T_ clusters designated based on expression of subset-associated transcripts (*CTSG* and *CMA1* for MC_TC,_ *IL17RB* and *GPR183* for MC_T_), a transitional cluster, and proliferating MCs (Figure 1, A and B, and Supplemental Figure 2) (3). All clusters were detected in all donors; however, the proliferating cluster exhibited high variability (Supplemental Figure 2, A and B). Ribosomal content was elevated in MC_T_1, while mitochondrial content was highest in transitional MCs (Supplemental Figure 2C).

Differential expression analysis indicated common core sets of transcripts distinguishing MC_TC_ (*CMA1*, *CTSG*, *FCER1A*, *GPR65*, *C3AR1*) from MC_T_ (*CPA3*, *TPSAB1*, *GPR183*, *CD38*, *IL17RB*) (Figure 1B). However, each MC_TC_ and MC_T_ subcluster was also associated with distinct transcriptional cassettes (Figure 1C and Supplemental Table 1). These transcripts included genes encoding granule factors, the γ subunit of FcεRI, and hematopoietic prostaglandin D_2_ (PGD_2_) synthase in MC_TC_1 (*FCER1G*, *NDST2*, *HPGDS*), inflammation-associated factors in MC_TC_2 (*CSF2*, *IL13*, *PTGS2*), ribosome and translation initiation factors in MC_T_1 (*RPL10A*, *RPS27*, *EIF3E*), a separate set of inflammation-associated factors in MC_T_2 (*PTGS1*, *LTC4S*, *IL5*), semaphorins and DNA-binding proteins in transitional cells (*SEMA4A*, *SEMA7A*, *POLG2*), and cell cycle–associated genes in the proliferating cluster (Figure 1C).

To gain further insight into the biological processes associated with each cluster, we conducted gene ontology (GO) pathway enrichment analysis (Supplemental Table 2). MC_TC_1 exhibited enrichment for electron transport chain and protein processing (Figure 1D), while MC_TC_2 was enriched in inflammation and adhesion-associated transcripts (Figure 1E). Transitional MCs were enriched for chromatin and histone modification and chromatin remodeling pathways, suggesting ongoing epigenetic reprogramming (Supplemental Figure 3), consistent with prior characterization of this population as immature MCs undergoing polarization (3).

MC_T_1 exhibited limited enrichment for biological processes, suggesting MC_T_-specific pathways are not well represented within the GO database. Among the pathways identified, MC_T_1 was enriched for translation and ribosome biogenesis (Figure 1F), whereas MC_T_2 showed enrichment for cell activation, the MHC-I pathway, and SMAD phosphorylation (Figure 1G). Several TGF-β target transcripts previously identified in murine inflammation-expanded airway MCs were significantly elevated in the transitional and both MC_T_ clusters (*SKIL*, *SMAD7*, and *LDLRAD4*), while *ITGAE* was restricted to the MC_T_ clusters (Supplemental Figure 2E) (11), suggesting a broader role for TGF-β signaling within the intraepithelial MC compartment.

### Intraepithelial MCs reside in a TGF-β–rich tissue niche.

MC_T_ enrichment for the SMAD phosphorylation pathway and murine MC TGF-β target genes was of particular interest based on our prior findings linking TGF-β to lower airway intraepithelial MC differentiation in vivo (11). To assess potential sources of TGF-β signaling in nasal polyps, we used a prior transcriptional atlas of sinonasal tissues, identifying basal epithelial cells (EpCs) as a predominant source for transcript encoding TGF-β2, while TGF-β1 was more broadly expressed (Supplemental Figure 4A) (4, 13). Reanalysis of a prior bulk RNA-Seq dataset of flow-sorted basal EpCs (13) indicated upregulation of both transcripts in subjects with CRSwNP (inclusive of AERD) relative to CRS without nasal polyps (CRSsNP), a milder clinical phenotype lacking an expanded MC_T_ population (Supplemental Figure 4B and Supplemental Table 3). Notably, although TGF-β2 binds the TGF-β receptor with lower affinity than TGF-β1, both isoforms direct human MC chemotaxis and inhibit their proliferation with similar potency (14, 15).

Transcript encoding integrin β_6_ (*ITGB6*), which pairs with integrin α_V_ to mediate latent TGF-β activation and regulate murine mucosal MC development (16, 17), showed a trend toward upregulation in CRSwNP EpC compared with CRSsNP (Supplemental Figure 4B). Flow cytometric evaluation identified a significant increase of cell-surface α_V_β_6_ on EpCs from both CRSwNP and AERD polyps relative to CRSsNP, suggesting increased availability of activated TGF-β within or adjacent to the epithelium in nasal polyps (Supplemental Figure 4C). Analysis of lung EpCs from a recent scRNA-Seq study similarly indicated enrichment of *TGFB1* and *TGFB2* in basal EpCs, which significantly upregulated both *ITGB6* and *ITGAV* in asthmatic patients but not allergic nonasthmatic patients following allergen challenge (Supplemental Figure 4D and Supplemental Table 4) (18).

To determine the functional consequences of EpC α_V_β_6_ expression, we conducted histologic assessment of SMAD2/3 phosphorylation within the epithelium, subepithelium, parenchyma, and glandular regions of tissue sections from CRSsNP and CRSwNP donors, normalized by cell density (Supplemental Figure 5). In CRSwNP, we observed significantly increased pSMAD2/3 in the epithelium relative to the parenchyma, with similar pSMAD2/3 levels in the epithelium and subepithelium (*P*_adj_ < 0.05). CRSsNP samples showed a trend toward elevated pSMAD2/3 in the epithelium and glandular regions of the tissue but no significant differences across compartments, with low phosphorylation in the subepithelium and parenchyma. These observations suggest that EpC α_V_β_6_ expression contributes to TGF-β signaling in proximity to the epithelium, indicating intraepithelial MCs reside within a TGF-β–rich tissue niche. Thus, together with our prior observations in mice, we hypothesized TGF-β signaling could direct the human intraepithelial MC_T_ phenotype.

### TGF-β directs an MC_T_-like transcriptional phenotype.

**Figure 2:**
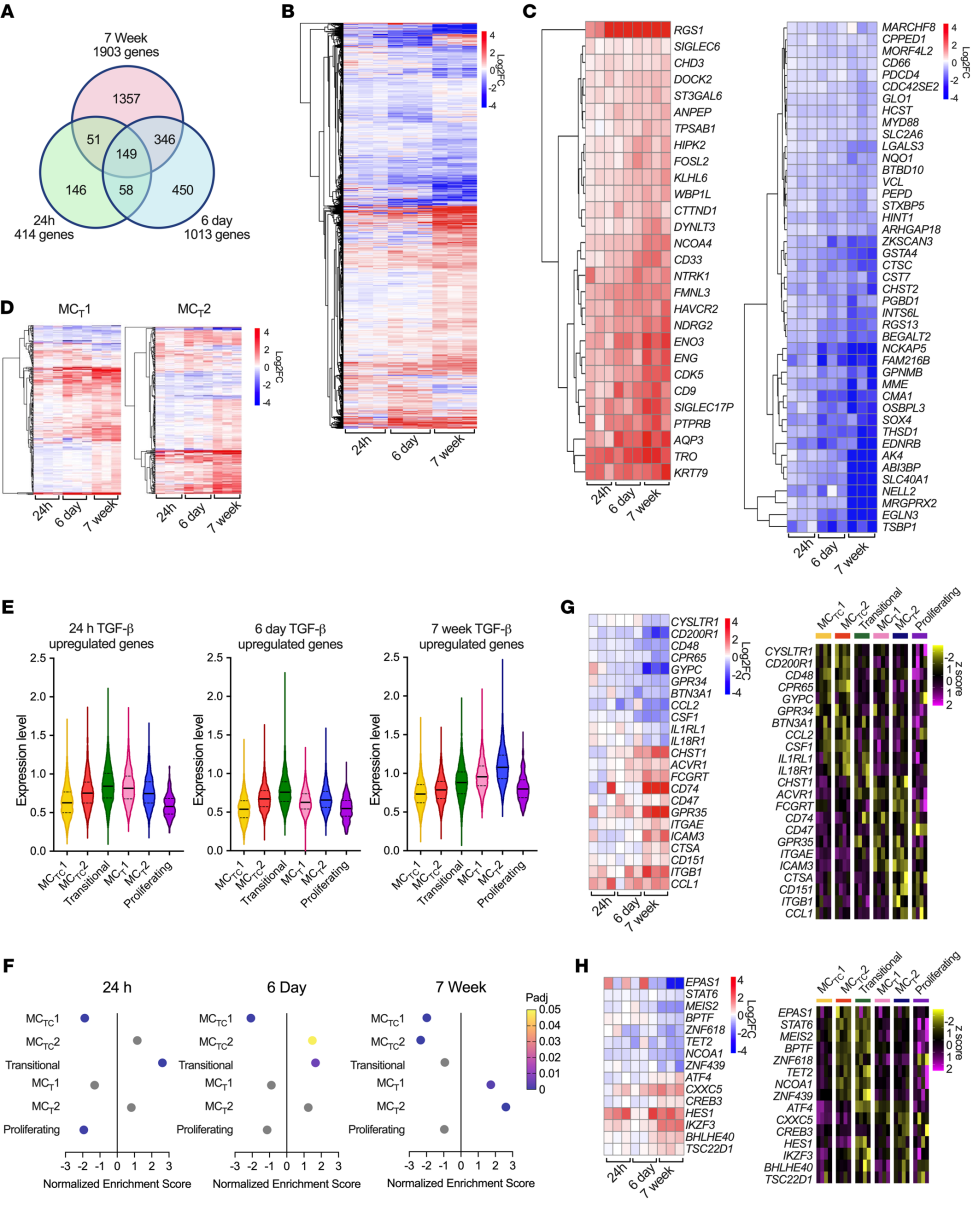
TGF-β signaling during MC development elicits an MC_T_-like transcriptional phenotype. (**A**) Venn diagram showing common versus timepoint-specific differentially expressed in PB-MC treated with TGF-β1 for 24 hours (green) and 6 days (blue) or PB-MCs differentiated in TGF-β1 for 7 weeks (pink) (FDR < 0.05, DESeq2). (**B** and **C**) Heatmap of (**B**) all differentially expressed genes and (**C**) transcripts showing gradients of upregulation (left) or downregulation (right) following TGF-β1 treatment (FDR < 0.05, DESeq2); color indicates log_2_fold change versus untreated samples. (**D**) Heatmap of differentially expressed genes associated with MC_T_1 (left) and MC_T_2 (right) clusters. (**E**) Violin plots showing per-cell expression as a percentage of all transcripts for timepoint-specific TGF-β1 target genes in PB-MCs treated with TGF-β1 for 24 hours, 6 days, or differentiated in TGF-β1 across AERD MC clusters (*P*_adj_ matrix shown in Supplemental Figure 6A). (**F**) GSEA for 7-week, 6-day, and 24-hour TGF-β in vitro signatures across AERD MC clusters, showing normalized enrichment score and adjusted *P* values. Gray color indicates statistically insignificant positive or negative enrichment. (**G** and **H**) Heatmap of (**G**) selected differentially expressed genes and (**H**) transcription factors both restricted to PB-MCs differentiated in TGF-β1 across time points (left) and differentially expressed across AERD MC clusters (right). Heatmaps show log_2_FoldChange versus untreated for each donor (bulk) or donor-averaged expression values (scRNA-Seq); scale bars show log_2_FoldChange versus untreated (bulk) or *z* score (scRNA-Seq).

To test the relationship between TGF-β signaling and the MC_T_ transcriptional phenotype, PB-MCs were treated for 24 hours or 6 days with TGF-β1 or, in separate experiments, MCs were differentiated from CD34^+^ cells in the presence or absence of TGF-β1 for the full duration of culture (7 weeks) and assessed via bulk RNA-Seq. TGF-β1 treatment exerted discrete, time-dependent effects on the MC transcriptome, differentially regulating 414 genes at 24 hours, 1,013 genes at 6 days, and 1,903 genes at 7 weeks (Figure 2, A and B, and Supplemental Table 5). A core set of 149 genes were significantly altered by TGF-β1 across all time points, including upregulation of *IL4R*, *SIGLEC6*, and *LTC4S* and downregulation of *CMA1*, *CTSG*, and *MRGPRX2*, with approximately half of the transcripts changing to a similar degree and the other half showing a gradient of upregulation or downregulation with time (Figure 2C). Transcripts enriched in the MC_T_1 and MC_T_2 clusters in vivo were predominantly upregulated in PB-MCs treated with or differentiated in TGF-β1 (Figure 2D).

We next constructed TGF-β–upregulated gene signatures unique to each time point to probe our in vivo dataset (Supplemental Table 5). The 24-hour and 6-day gene signatures showed low-level expression across MC clusters, with lowest expression in the MC_TC_1 and proliferating clusters but only modest differences between the remaining MC clusters (Figure 2E, *P* value matrix in Supplemental Figure 6A). The 7-week developmental TGF-β1 signature had substantially higher expression in both MC_T_ clusters and the transitional cluster with significant elevation relative to both MC_TC_ clusters (Figure 2E). The core TGF-β1 target genes included MC-restricted proteases and cell-surface receptors (Figure 2C), indicating a cell type–specific effect on human MCs and potentially explaining the limited detection of TGF-β signaling within the MC_T_ clusters through pathway enrichment (Figure 1, F and G). When we instead used our MC-derived gene signatures for gene set enrichment analysis (GSEA), we identified significant elevation of the 7-week signature in both MC_T_ clusters and significant decreases in the MC_TC_ clusters (Figure 2F and Supplemental Table 2). The 6-day signature was significantly enriched in the transitional and MC_TC_1 clusters and decreased in MC_TC_2, while only the transitional cluster was enriched for the 24-hour signature (*P*_adj_ < 0.05).

Notably, a subset of the genes differentially expressed between MC_T_ and MC_TC_ clusters in vivo were only significantly altered in PB-MCs differentiated in TGF-β1, including several MC_T_-enriched transcription factors (*HES1*, *AEBP1*, *IKZF3*) (Figure 2, G and H). PB-MC differentiation in TGF-β further drove upregulation of transcripts encoding ribosomal components, mirroring MC_T_ ribosomal enrichment in vivo (Supplemental Figure 6B). CRSwNP MC_T_ and MC_TC_ exhibited similar cell-surface expression of the TGF-β receptor R2 subunit (TGF-β R2), which pairs with the R1 subunit to recognize TGF-β1 and TGF-β2 (Supplemental Figure 6, C and D). As additional controls, we confirmed that flow sorting did not elicit degranulation or impact viability in PB-MC_T_ or PB-MC_TC_ (Supplemental Figure 6E). Together with the pSMAD2/3 patterns in CRSwNP, these observations strongly suggested that differences in developmentally associated exposure to TGF-β across tissue microenvironments direct MC polarization in vivo and potentially implicated shorter-term TGF-β signaling as a factor differentiating MC_TC_1 and MC_TC_2 clusters.

To explore whether the TGF-β developmental signature was restricted to MC_T_s in AERD or represented a broader feature of MC_T_s, we expanded our analysis to other diseases and tissues. We first assessed our prior sinonasal polyp MC dataset (3), finding that MC_T_s in both AERD and aspirin-tolerant CRSwNP were enriched for the TGF-β1 developmental signature relative to MC_TC_s (Supplemental Figure 6F). We next evaluated MCs from an scRNA-Seq study of ulcerative colitis that fractionated the intestinal epithelium from lamina propria (19). MCs from the lamina propria fraction were significantly enriched for core sinus MC_TC_ transcripts (*CMA1*, *GPR65*, *ICAM1*) and expressed inflammation-associated sinus MC_TC_2 transcripts (*PTGS2*, *CSF1*) (FDR < 0.05). Expression of *IL13* was only detected in the lamina propria, although this did not reach statistical significance (Supplemental Figure 6G). MCs from the epithelial fraction were significantly enriched for sinus MC_T_ core transcripts (*TPSAB1*, *CTSW*, *CD9*) and for sinus MC_T_1-enriched transcripts encoding eicosanoid biosynthetic enzymes (*PTGS1*, *LTC4S*, *ALOX5*) (FDR < 0.05). Epithelial MCs further exhibited significant enrichment for the TGF-β developmental signature compared with lamina propria MCs (Supplemental Figure 6H and Supplemental Table 6). Collectively, these observations supported a role for TGF-β in directing the MC_T_ transcriptome across mucosal tissues and suggested a link between TGF-β developmental signaling and MC effector function.

### Defining the influence of TGF-β on the MC granule phenotype.

**Figure 3:**
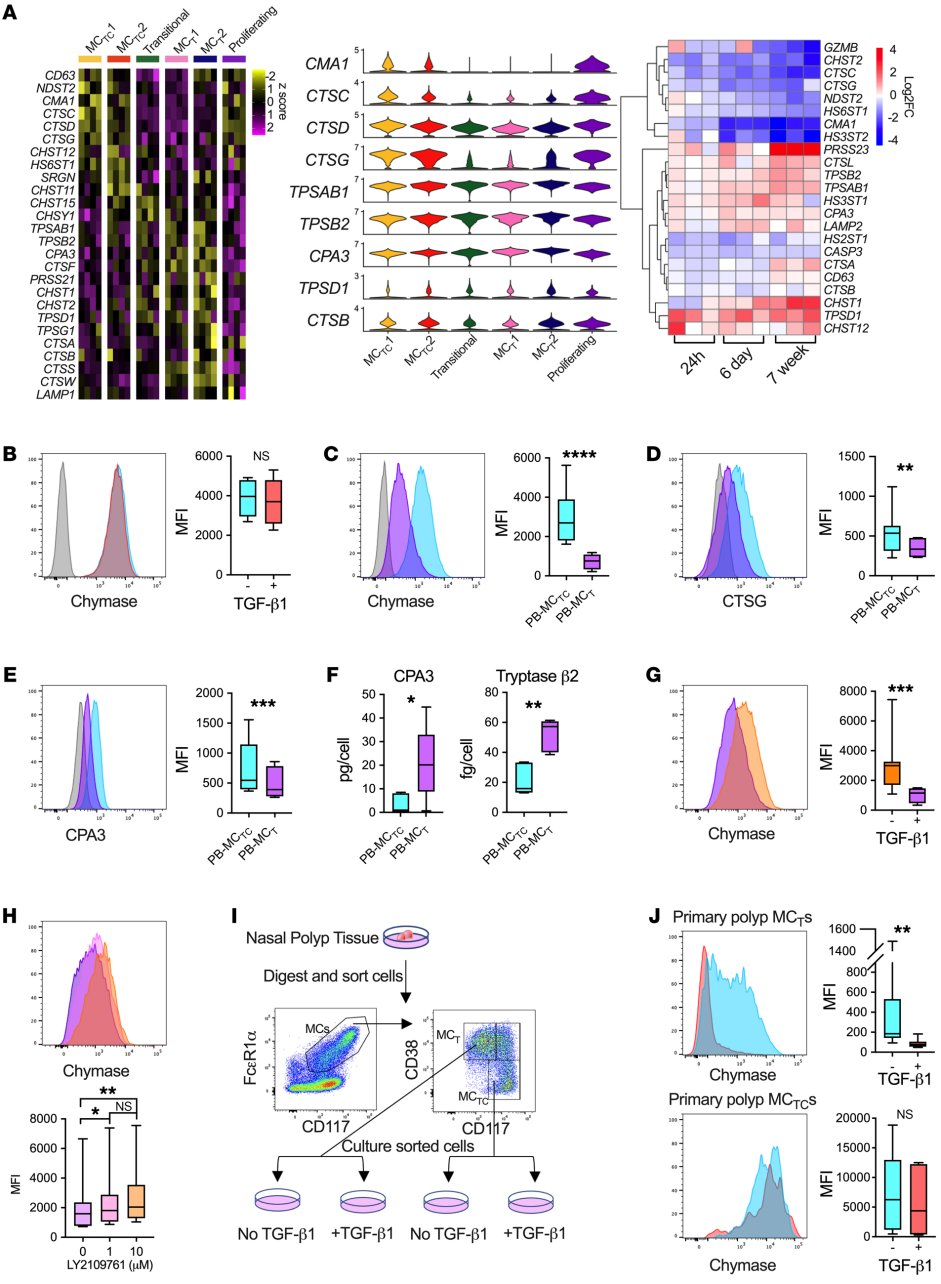
TGF-β signaling directs the MC_T_ protease phenotype during early development. (**A**) Differentially expressed genes encoding granule components in vivo (left), with violin plots for select proteases (center), and in vitro gene expression for PB-MC stimulated with TGF-β1 for 24 hours, 6 days, or differentiated in TGF-β1 (right). Columns indicate donor-averaged cluster expression (scRNA-Seq) or log_2_FoldChange versus untreated for each donor (bulk); scale bars denote *z* score (scRNA-Seq) or log_2_FoldChange (bulk), FDR < 0.05, log_2_FoldChange > 0.5 for scRNA-Seq and FDR < 0.05 for bulk (DESeq2). (**B**) Chymase expression and quantification in PB-MCs treated with (red) or without (blue) TGF-β1 for 6 days versus isotype control (gray). *n* = 6 individual donors (*t* test). (**C**–**E**) Expression and quantification of (**C**) chymase, (**D**) CTSG, and (**E**) CPA3 in PB-MCs differentiated with (purple) or without (blue) TGF-β1 versus isotype control (gray). *n* = 7–8 individual donors. **P < 0.01; ***P < 0.001; ****P < 0.0001 (*t* test). (**F**) One-week CPA3 and tryptase β2 release in PB-MC_T_ versus PB-MC_TC_ supernatants. *n* = 6 and 5 individual donors, respectively. *P < 0.05; **P < 0.01 (Mann-Whitney). (**G**) Chymase expression and quantification for PB-MCs differentiated in TGF-β1 and subsequently cultured with (purple) or without (orange) TGF-β1 for 2 weeks. *n* = 8 individual donors. ***P < 0.001 (*t* test). (**H**) Chymase expression and quantification for PB-MC_T_ cocultured with EpCs for 2 weeks supplemented with SCF (100 ng/mL), IL-6 (50 ng/mL), and the indicated concentration of LY2109761. *n* = 8. *P_adj_ < 0.05, **P_adj_ < 0.01 (ANOVA) (**I**) Gating strategy to isolate nasal polyp MC_T_s and MC_TC_s. (**J**) Chymase expression and quantification for primary nasal polyp MC_T_s (top) or MC_TC_s (bottom) maintained in culture media with (red) or without (blue) TGF-β1 for 2 weeks. *n* = 6–7 for nasal polyp MCs. **P < 0.01 (*t* test). Box-and-whisker plots show median, interquartile range, and minimum/maximum values observed.

As the MC_TC_-associated proteases *CMA1* and *CTSG* (encoding chymase and CTSG, respectively) were strongly downregulated by TGF-β treatment at all 3 time points (Figure 2C and Supplemental Figure 7A), we next assessed granule-associated transcripts. Nasal polyp MC_T_s were enriched for *CHSY1*, encoding chondroitin sulfate synthase, genes encoding tryptases (*TPSAB1*, *TPSB2*, *TPSD1*, *TPSG1*), *CPA3*, encoding carboxypeptidase A3, and *PRSS21*, a serine protease not previously associated with MCs, suggesting a greater degree of protease heterogeneity in human MCs than previously appreciated (Figure 3A). MC_TC_s were enriched for *NDST2*, encoding a central enzyme required for synthesis of heparin sulfate, a major proteoglycan component of MC_TC_ granules (20, 21), *CMA1*, and several cathepsin-encoding transcripts (*CTSC*, *CTSD*, *CTSG*). TGF-β drove similar expression patterns to those observed in vivo, downregulating *CMA1*, *CTSG*, and *NDST2* while upregulating transcripts encoding CPA3 and α, β, and δ tryptases (*TPSAB1*, *TPSB2*, *TPSD1*) (Figure 3A and Supplemental Figure 7A). Thus, TGF-β profoundly altered expression of transcripts regulating MC granule composition and protease content.

PB-MC differentiated in stem cell factor (SCF) and IL-6 alone contained high levels of intracellular chymase, indicative of an MC_TC_-like protease phenotype content (PB-MC_TC_) (Figure 3B). Intracellular chymase content was unaffected by a 6-day TGF-β1 treatment despite the striking change in transcript expression, indicating a potential “uncoupling” of the MC transcriptional and protease phenotype. We hypothesized that chymase stability was due to slow turnover of MC granule–associated proteins. Supporting this, PB-MC differentiation in TGF-β1 significantly reduced intracellular chymase content (Figure 3C), directing an MC_T_-like protease phenotype (PB-MC_T_). PB-MC differentiated in TGF-β1 further exhibited significant reductions in intracellular CTSG (Figure 3D), a protease robustly expressed by MC_TC_ in vivo but absent from MC_T_ (Supplemental Figure 8). While *CPA3* was transcriptionally upregulated by TGF-β1 (Figure 3A), intracellular CPA3 protein was lower in PB-MC_T_s relative to PB-MC_TC_s (Figure 3E). Instead, we observed significant elevations in extracellular CPA3 in PB-MC_T_ culture supernatants relative to PB-MC_TC_ (Figure 3F). Tryptase β2, encoded by *TPSB2*, was similarly elevated in PB-MC_T_ supernatants (Figure 3F). In stark contrast to the stability of the PB-MC_TC_ protease phenotype, intracellular chymase content of PB-MC_T_ significantly increased following TGF-β1 removal (Figure 3G). Coculture with EpCs maintained the PB-MC_T_ chymase-low phenotype in the absence of exogenous TGF-β, while treatment of cocultures with the TGF-β receptor kinase inhibitor LY2109761 increased intracellular chymase in a dose-dependent manner (Figure 3H), further implicating EpC-derived TGF-β as responsible for maintaining the MC_T_ protease phenotype in vivo.

Following our observations with in vitro–differentiated PB-MC_T_ and PB-MC_TC_, primary MC_T_s and MC_TC_s were flow-sorted from nasal polyps using previously defined surface markers (Figure 3I) and cultured for 2 weeks supported by SCF either alone or supplemented with TGF-β1 (3). Consistent with in vitro–differentiated MCs, primary tissue MC_T_s maintained a chymase-low phenotype in TGF-β–supplemented media, while culture in SCF alone led to increased chymase content (Figure 3J). In contrast, the protease phenotype of primary MC_TC_s was highly stable, with TGF-β supplementation having no significant impact on chymase content (Figure 3J). TGF-β signaling during MC differentiation was required to establish the MC_T_ protease phenotype, while interruptions in TGF-β1 signaling increased intracellular chymase levels in both PB-MC_T_s and polyp MC_T_s. Thus, our in vitro and ex vivo findings indicate a 1-way MC granular plasticity, with MC_TC_s having a stable granule phenotype but MC_T_s increasing intracellular chymase content if TGF-β signaling is interrupted. These observations further suggest that chymase expression is a “default” pathway for MCs and must be actively suppressed to maintain the MC_T_ phenotype.

### TGF-β regulation of MC surface receptor expression.

**Figure 4:**
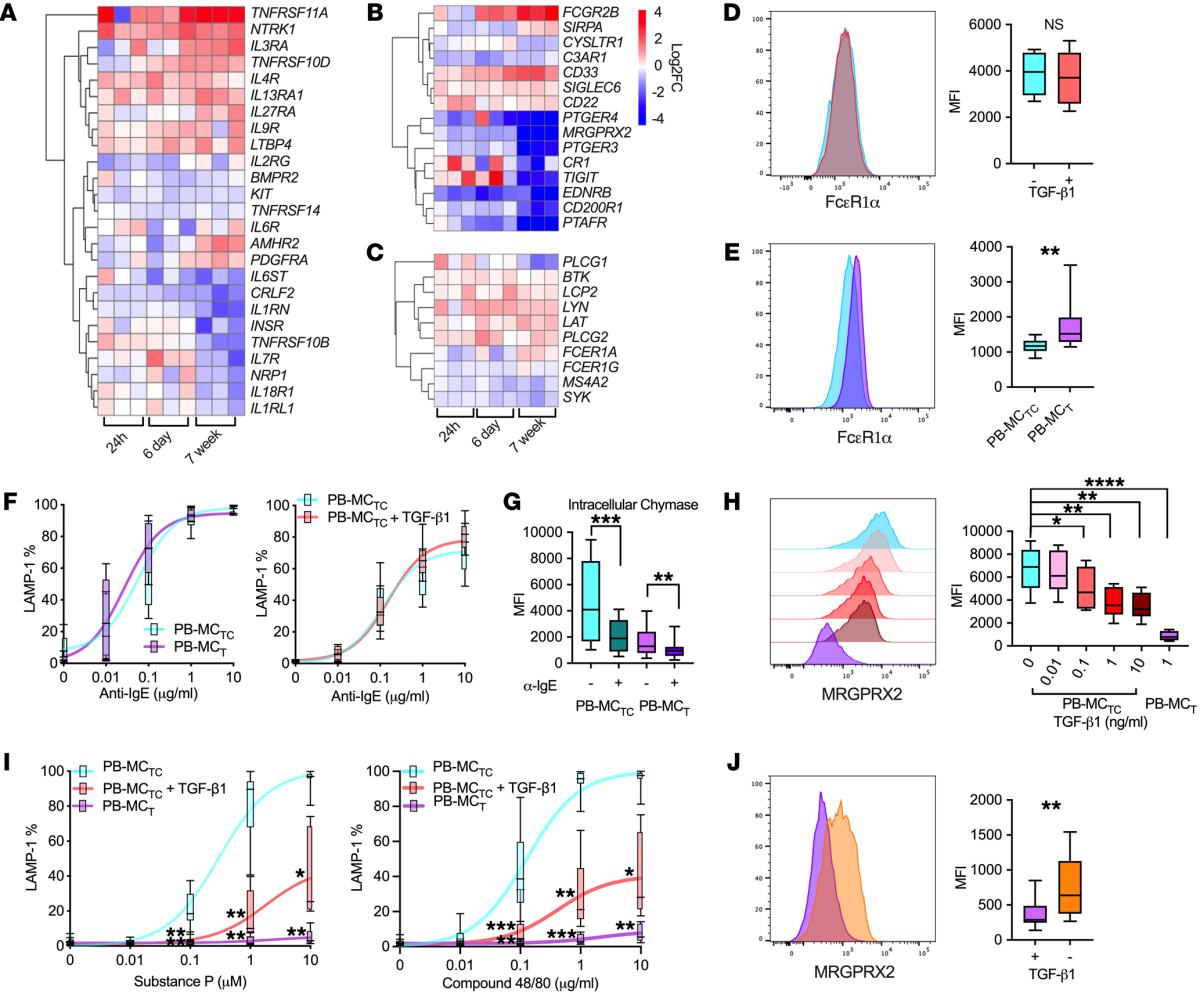
TGF-β selectively regulates MC expression of activating receptors. (**A**–**C**) Heatmaps of select differentially expressed transcripts encoding (**A**) cytokine, chemokine, and growth factor receptors, (**B**) activating and inhibitory receptors, and (**C**) FcɛR1 signaling pathway components following PB-MC stimulation with TGF-β1 for 24 hours or 6 days or differentiation of MCs in TGF-β1 for 7 weeks. Heatmaps show log_2_FoldChange expression of genes versus unstimulated cells at each time point. Columns indicate individual donors, FDR < 0.05 (DESeq2). (**D**) Representative flow plot and quantification of FcɛR1α expression by PB-MC_TC_s treated with (red) or without (blue) TGF-β1 for 6 days. *n* = 12 (*t* test). (**E**) Expression and quantification of FcɛR1α in PB-MC_T_s (purple) versus PB-MC_TC_s (blue). *n* = 8. **P < 0.01 (*t* test). (**F**) Degranulation of PB-MC_TC_s treated with or without TGF-β1 for 6 days (left) or PB-MC_T_s (right) at 1 hour after activation with anti-IgE. *n* = 5 individual donors (ANOVA). (**G**) Intracellular chymase content of PB-MC_TC_ (light blue) and PB-MC_T_ (purple) at baseline and following degranulation with anti-IgE. *n* = 7 individual donors. **P_adj_ < 0.01; ***P_adj_ < 0.001 (ANOVA). (**H**) MRGPRX2 expression and quantification for PB-MC_TC_s following 6-day treatment with (red gradient) or without (blue) TGF-β1 and PB-MC_T_s (purple). *n* = 8 individual donors. **P_adj_ < 0.01; ***P_adj_ < 0.001; ****P_adj_ < 0.0001 (ANOVA). (**I**) Degranulation of PB-MC_TC_s treated with (red) or without (blue) TGF-β1 for 6 days, and PB-MC_T_s (purple) following 1 hour stimulation with MRGPRX2 ligands compound 48/80 (left) and substance P (right). *n* = 5–6 individual donors. **P_adj_ < 0.01; ***P_adj_ < 0.001; ****P_adj_ < 0.0001 (ANOVA). (**J**) Representative flow plot and quantification of MRGPRX2 expression by PB-MC_T_s maintained in culture media with (purple) or without (orange) TGF-β1 for 2 weeks. *n* = 8 individual donors. **P < 0.01 (*t* test). Box-and-whisker plots show median, interquartile range, and minimum/maximum values observed.

MCs express a broad repertoire of cell surface receptors responsible for fine tuning their responses to microenvironmental cues. Across sinonasal MCs, we observed differential expression of cytokine and growth factor receptors (*KIT*, *CSF2RB*, *IL17RB*, *NTRK1*) and receptors associated with MC activation or inhibition (*FCER1A*, *C3AR1*, *CD33*, *SIGLEC6*) (Supplemental Figure 9A). TGF-β1 treatment in vitro influenced a subset of these receptors, upregulating transcripts encoding receptors for IL-3 (*IL3RA*) and IL-9 (*IL9R*), which can regulate MC growth and proliferation, and *NTRK1*, encoding a receptor for nerve growth factor (Figure 4A and Supplemental Figure 7B), a growth factor elevated in sinus mucosa and asthmatic bronchial epithelium (22–24). TGF-β1 upregulated expression of the α subunit of the receptor for IL-4 (*IL4R*) at all time points, a signal previously found to drive ex vivo proliferation of human intestinal MC_T_ (25). Further, TGF-β1 upregulated inhibitory receptors (*CD22*, *CD33*, *SIGLEC6*, *FCGR2B*) while downregulating the transcripts encoding IL-1 family cytokine receptors (*IL18R1*, *IL1RL1*, *IL1RN*) and receptors linked with IgE-independent MC degranulation, including the platelet-activating factor receptor (*PTAFR*), the complement component C3a receptor (*C3AR1*), and *MRGPRX2*, which mediates MC degranulation in response to neuron-derived substance P and during pseudoallergic drug reactions (Figure 4B) (26–28). Many of these receptors were differentially expressed between polyp MC_T_ and MC_TC_ clusters in vivo, including enrichment of *SIGLEC6* and *NTRK1* in MC_T_s and *IL18R1*, *IL1RL1*, *PTAFR*, and *C3AR1* in MC_TC_s (Supplemental Figure 9A).

TGF-β1 treatment impacted several transcripts associated with the high-affinity IgE receptor signaling pathway, including upregulating transcripts encoding the FcεR1 α chain (*FCER1A*) while downregulating transcripts encoding the β chain (*MS4A2*). TGF-β1 further influenced key downstream signaling pathway components, downregulating *SYK* and *PLCG1* while upregulating *LAT*, *LYN*, and *PLCG2* (Figure 4C and Supplemental Figure 7B). Despite prior reports of TGF-β1 downregulating murine MC FcεR1α expression and inhibiting IgE-mediated activation (29, 30), TGF-β1 treatment did not alter FcεR1α surface expression on PB-MC_TC_ (Figure 4D and Supplemental Figure 9B), whereas FcεR1α was moderately elevated on PB-MC_T_s relative to PB-MC_TC_ (Figure 4E). No differences were observed in IgE/anti-IgE-driven degranulation between either PB-MC_T_ and PB-MC_TC_ or PB-MC_TC_s treated with or without TGF-β1 (Figure 4F and Supplemental Figure 9C). However, intracellular staining for chymase indicated a significantly larger MFI reduction in PB-MC_TC_s (2938 ± 844.7) compared with PB-MC_T_ (719.3 ± 182.5) (Figure 4G). Thus, although degranulation levels were similar between the 2 subsets, due to differences in granule composition, the mediators released during the degranulation process likely differ considerably.

As MRGPRX2 is a well-established driver of MC_TC_ degranulation (31), we evaluated MRGPRX2 surface expression and response to its ligands. TGF-β1 treatment moderately decreased PB-MC_TC_ surface expression of MRGPRX2 at 24 hours, while substantial dose-dependent downregulation was observed at 6 days (Supplemental Figure 9D and Figure 4H). PB-MC_T_s displayed minimal MRGPRX2 expression (Figure 4H). Consequently, PB-MC_TC_ degranulation in response to activation with the MRGPRX2 ligands compound 48/80 and substance P was significantly reduced in a dose-dependent manner following 6-day stimulation with TGF-β1, while degranulation was abolished in PB-MC_T_s (Figure 4I). As with intracellular chymase content, removal of TGF-β1 from PB-MC_T_ culture media for 2 weeks increased MRGPRX2 expression (Figure 4J). Collectively, these findings establish that MRGPRX2 protein expression and activity are dynamically regulated by TGF-β, which may have a similar effect on other IgE-independent MC activating receptors. Further, in contrast to the MC_TC_ granule phenotype, these observations indicate that the MC_TC_ cell-surface phenotype exhibits substantial plasticity.

### TGF-β facilitates a distinctive MC_T_ effector phenotype.

**Figure 5:**
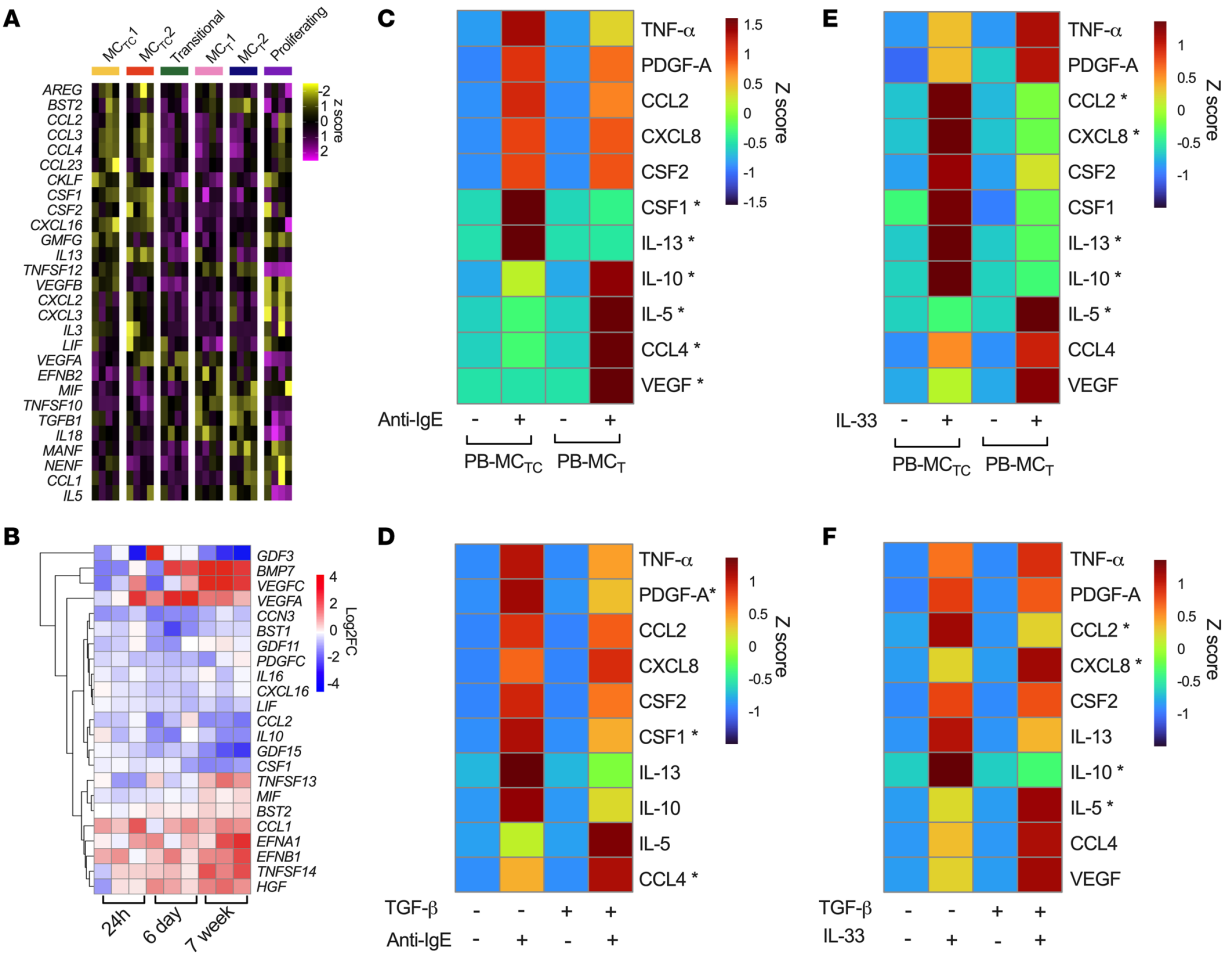
TGF-β selectively reshapes MC proinflammatory cytokine, chemokine, and growth factor production following IgE crosslinking. (**A**) Row-normalized heatmap of differentially expressed genes associated with cytokine, chemokine, and growth factors across nasal polyp MC clusters. Columns show averaged expression by donor; scale bar denotes *z* score. FDR < 0.05, log_2_FoldChange > 0.5 (DESeq2). (**B**) Heatmap showing differentially expressed transcripts in TGF-β1–stimulated cells. Columns show individual donors; scale bars indicate log_2_FoldChange versus unstimulated controls. FDR < 0.05 (DESeq2) (**C** and **D**) Row-normalized average data of *n* = 6 individual donors showing protein secretion of cytokines, chemokines, and growth factors at 6 hours following anti-IgE activation by (**C**) PB-MC_TC_s versus PB-MC_T_s and (**D**) PB-MC_TC_s cultured with or without TGF-β1 for 6 days. (**E** and **F**) Row-normalized average data of *n* = 6 individual donors showing release of cytokines, chemokines, and growth factors at 6 hours after IL-33 stimulus by (**E**) PB-MC_TC_s versus PB-MC_T_s and (**F**) PB-MC_TC_s cultured with or without TGF-β1 for 6 days. Scale bars denote *z* score. *P_adj_ < 0.05 between activated PB-MC_T_ and PB-MC_TC_ (ANOVA).

MC effector functions are mediated by both release of preformed mediators and de novo production of protein and lipid mediators. Extending our prior observations, we noted differential expression of many cytokine and chemokine transcripts across polyp MC subsets in vivo. MC_TC_s showed elevated expression of chemokines for monocytes and/or T cells (*CCL2*, *CCL4*, *CCL23*, *CXCL16*), the cytokine *IL13*, and the monocyte/macrophage lineage-associated growth factors *CSF1* and *CSF2*. MC_TC_2 additionally expressed neutrophil chemokines (*CXCL2*, *CXCL3*), several cytokines (*IL3*, *LIF*), and the growth factor *VEGFA* (Figure 5A and Supplemental Figure 10). All MC_T_s were transcriptionally enriched for a separate set of cytokines (*IL18*, *MIF*, *TGFB1*, *TNFSF10*) (Figure 5A), while MC_T_2 were further enriched for *IL5* and *CCL1*. Thus, MC subsets within human nasal polyps likely carry out distinct microenvironmental-associated effector functions, potentially including regional recruitment and retention of other leukocyte subsets.

Following our initial characterization of TGF-β1 regulation of the MC granule and surface phenotypes, we hypothesized it could further direct the discrete cytokine, chemokine, and growth factor profiles enriched in nasal polyp MC_T_s in vivo. PB-MC_T_ downregulated a subset of MC_TC_-associated transcripts (*LIF*, *CXCL16*, *CCL2*, *CSF1*) and upregulated MC_T_-associated ones (*MIF*, *CCL1*) (Figure 5B and Supplemental Figure 7C). PB-MC_TC_ treatment with TGF-β1 had minimal impact on inflammatory mediators. Transcripts encoding cytokines such as IL-5 and IL-13 are typically upregulated in response to MC activation (32). Thus, we examined whether TGF-β1 modified activation-induced MC mediator production by activating PB-MC_T_ and PB-MC_TC_ with IL-33 or IgE crosslinking. PB-MC_TC_s treated with TGF-β1 for 6 days were evaluated in parallel. Minimal baseline secretion was observed for any mediators measured, despite constitutive transcript expression for several (*CCL2*, *CSF1*, *VEGFA*, *IL10*) (Supplemental Figures 11 and 12). Following IgE cross-linking, PB-MC_T_s secreted significantly elevated levels of IL-5, IL-10, CCL4, and VEGF, while PB-MC_TC_s preferentially secreted IL-13 and CSF1 (M-CSF) (*P*_adj_ < 0.05). No significant differences were observed in TNF-α, PDGF-A, CCL2, CSF2 (GM-CSF), or CXCL8 (IL-8) secretion following IgE crosslinking. PB-MC_TC_ treatment with TGF-β for 6 days increased CCL4 secretion while reducing CSF1 and PDGF-A, but no significant differences were observed for IL-5, IL-10, or IL-13 (Figure 5, C and D, and Supplemental Figure 11B).

In response to IL-33 treatment, PB-MC_T_s again produced significantly more IL-5, while PB-MC_TC_s preferentially produced IL-13 (Figure 5E and Supplemental Figure 12A). In contrast to IgE crosslinking, following IL-33 activation, PB-MC_T_s produced significantly less IL-10, IL-8, and CCL2 (Figure 5E and Supplemental Figure 12A). No significant differences were observed between PB-MC_T_s and PB-MC_TC_s for CCL4, VEGF, CSF1, PDGF-A, or TNF-α following IL-33 stimulus (Figure 5E and Supplemental Figure 12A). Six-day treatment of PB-MC_TC_ with TGF-β1 significantly increased IL-5 and CXCL8 secretion, while inhibiting production of IL-10 and CCL2, but again had no impact on IL-13 production (*P*_adj_ < 0.05) (Figure 5F and Supplemental Figure 12B). Notably, the PB-MC_T_ mediator secretion pattern following IL-33 activation (enhanced IL-5 with reduced IL-13 and CCL2) mirrored the differences in transcripts encoding these T2-associated factors in polyp MC_T_s versus MC_TC_s (Figure 5A), while the secretion pattern following the 6-day stimulation (increased IL-5 but no difference in IL-13 or CSF2) was suggestive of the differential expression patterns observed between MC_TC_2 and MC_TC_1. Moreover, IL-33 was far more potent than IgE crosslinking for driving cytokine production, while IgE crosslinking drove increased chemokine secretion (Supplemental Figures 11 and 12). Overall, this suggested a major role for TGF-β in shaping MC cytokine and chemokine production following activation by diverse stimuli in vivo.

### Enhanced eicosanoid production by MC_T_.

**Figure 6:**
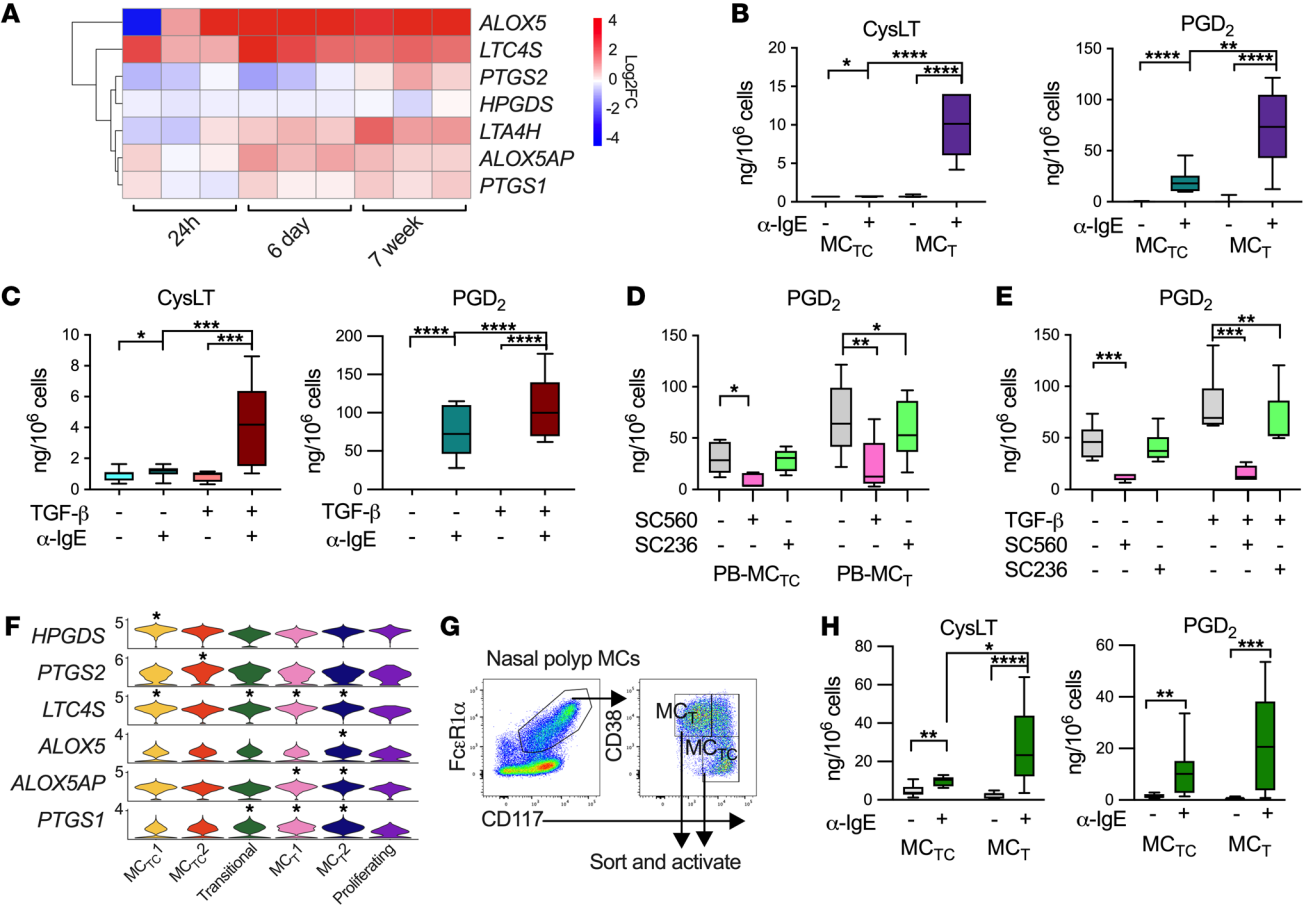
TGF-β enhances MC lipid mediator production. (**A**) Heatmap showing genes associated with eicosanoid biosynthesis differentially regulated by TGF-β1. *n* = 3 individual donors. FDR < 0.05, log_2_FoldChange > 0.5 (DESeq2). Scale bar indicates log_2_FoldChange versus unstimulated control samples. (**B**) CysLTs (left) and PGD_2_ (right) production by PB-MC_TC_s versus PB-MC_T_s and activated with anti-IgE for 1 hour. *n* = 8–9 individual donors. *P_adj_ < 0.05; **P_adj_ < 0.01; ****P_adj_ < 0.0001 (ANOVA). (**C**) CysLTs (left) and PGD_2_ (right) production by PB-MC_TC_s treated with or without TGF-β1 for 6 days and activated with anti-IgE for 1 hour. *n* = 15 individual donors. * *P*_adj_ < 0.05; *** *P*_adj_ < 0.001; ****P_adj_ < 0.0001 (ANOVA). (**D** and **E**) Effects of selective inhibitors for COX-1 (SC560) and COX-2 (SC236) on PGD_2_ production by (**D**) PB-MC_TC_s versus PB-MC_T_s or (**E**) PB-MC_TC_s treated with TGF-β1 for 6 days prior to activation with anti-IgE. *n* = 6 individual donors. * *P*_adj_ < 0.05; ** *P*_adj_ < 0.01; *** *P*_adj_ < 0.001 (ANOVA). (**F**) Violin plots of differentially expressed genes associated with CysLTs and PGD_2_ biosynthesis across polyp MC clusters, FDR< 0.05, log_2_FoldChange > 0.5 (DESeq2). (**G**) Gating strategy to isolate nasal polyp MC_T_ and MC_TC_s (left). (**H**) Eicosanoid production following activation with anti-IgE for 1 hour (right). *n* = 9 individual donors. * *P*_adj_ < 0.05; **P_adj_ < 0.01; *** *P*_adj_ < 0.001; ****P_adj_ < 0.0001 (ANOVA). Box-and-whisker plots show median, interquartile range, and minimum/maximum values observed.

As activated MCs are well-characterized sources of arachidonic acid metabolites, we evaluated the impact of TGF-β on eicosanoid biosynthetic enzyme expression. Notably, both at 6 days and 7 weeks, TGF-β1 upregulated the expression of transcripts encoding 5-lipoxygenase (*ALOX5*), 5-lipoxygenase activating protein (*ALOX5AP*), and leukotriene C_4_ synthase (*LTC4S*), proteins required for LTC_4_ synthesis (Figure 6A and Supplemental Figure 7D). *PTGS1*, encoding the enzyme cyclooxygenase (COX) 1, a key component of PGD_2_ production, was upregulated at both 6 days and 7 weeks (Figure 6A and Supplemental Figure 7D), while a trend toward *PTGS2* upregulation (encoding COX-2) was also observed at 7 weeks (FDR < 0.1). Thus, we hypothesized that TGF-β treatment would enhance MC eicosanoid production.

Consistent with their increased expressions of *ALOX5*, *ALOX5AP*, *LTC4S*, and COX enzymes, PB-MC_T_s produced significantly more cysteinyl leukotrienes (CysLTs) and PGD_2_ than PB-MC_TC_s following IgE crosslinking (Figure 6B), while 6-day treatment of PB-MC_TC_s with TGF-β1 also increased secretion of both eicosanoids (Figure 6C). As COX-1 and COX-2 can both convert arachidonic acid into PGH_2_, the precursor of PGD_2_, we assessed the role of each enzyme in driving the increased PGD_2_ production observed in vitro. Treatment of PB-MC_TC_s with the COX-1–specific inhibitor SC560 significantly reduced FcεR1-induced PGD_2_ synthesis in both groups (Figure 6, D and E), indicating a central role for this enzyme in driving PGD_2_ production, while the COX-2-specific inhibitor SC236 marginally decreased PGD_2_ production only in TGF-β1–treated cells (Figure 6, D and E). Together, these findings suggest that MC_T_s are poised for rapid PGD_2_ synthesis primarily due to TGF-β1–driven COX-1 upregulation.

Mirroring the effects of TGF-β in vitro, both in vivo MC_T_ clusters were enriched for *LTC4S*, *ALOX5AP*, and *PTGS1*, with MC_T_2 further enriched for *ALOX5* (Figure 6F). MC_TC_1 was enriched for *HPGDS,* encoding hematopoietic PGD_2_ synthase, while MC_TC_2 was further enriched for *PTGS2*. To test the functional consequences of these differential expression patterns, primary MC_TC_s and MC_T_s were flow sorted from nasal polyps (Supplemental Figure 6C and Figure 6G) and activated using anti-IgE. As with their in vitro counterparts, activated MC_T_s generated significantly higher quantities of CysLTs compared with MC_TC_s and trended toward generating more PGD_2_ than MC_TC_s on a per-cell basis (Figure 6H). Thus, our findings highlight MC_T_s as a dominant source of CysLT production both in vitro and in vivo, underscoring the discrete effector functions for MC_T_s and MC_TC_s, and identify TGF-β1 as the likely driver of the MC_T_ effector phenotype.

## Discussion

MCs in vitro preferentially develop into chymase-expressing cells under many culture systems, and key cytokines required for MC development and maturation, such as SCF, IL-6, and IL-4, can further induce chymase (33, 34). While airway EpC coculture suppresses chymase in cord blood–derived human MCs, the responsible factor was not identified (35). Here, we demonstrate that TGF-β1 can elicit MC_T_ differentiation in vitro, allowing direct study of the discrete functions of MC_TC_s and MC_T_s, and further identify TGF-β as a key regulator of MC chymase during EpC coculture. Whereas PB-MC_TC_ treatment with TGF-β1 elicited a partial MC_T_-like transcriptional phenotype (Figure 2), MCs differentiated in TGF-β1 more strongly resembled in vivo MC_T_ transcriptionally and uniquely acquired the MC_T_ protease phenotype (Figure 2 and Figure 3). Within sinus tissue, MC_T_s were enriched for the TGF-β1 developmental signature in both AERD and CRSwNP, while in the colon, MC from the epithelial fraction showed significant enrichment relative to those from the lamina propria (Supplemental Figure 6), together suggesting a broad role for TGF-β in directing MC_T_ differentiation across tissues and diseases. In addition to protease content, PB-MC_T_s mirror in vivo MC_T_ transcription factor expression and inflammatory mediator production capacity (Figure 2 and Figure 6). Thus, our findings carry potential major implications for understanding the role of TGF-β and MC_T_s in disease pathogenesis.

Although commonly thought of as an antiinflammatory cytokine, an emerging body of literature suggests a pathobiologic role for TGF-β in mucosal T2 inflammation (36, 37), at least partially due to its effects on innate immune effector cells (38, 39). TGF-β is initially secreted in a latent form that is deposited in the extracellular matrix and requires activation prior to signaling (40). One such activator, the α_V_β_6_ integrin heterodimer, is upregulated on damaged bronchial EpCs, which also leads to increased TGF-β secretion (41–43). Notably, this integrin pair was significantly elevated on EpC from nasal polyp tissue relative to nonpolyp control tissue, while acute allergen challenge drove induction of transcripts encoding both chains on EpCs in allergic asthmatics (Supplemental Figure 4). Consequently, SMAD2/3 phosphorylation was significantly higher in CRSwNP epithelium relative to polyp parenchyma (Supplemental Figure 5). Thus, the mucosal epithelium in T2-high disease is likely a particularly favorable niche for MC_T_ development.

TGF-β is elevated in the airways of asthmatics (44–46), while intraepithelial MC burden correlates with TGF-β levels in chronic obstructive pulmonary disease patients (47, 48). In seasonal rhinitis, nasal mucosal immunostaining for all TGF-β isoforms but especially TGF-β2 is elevated, while TGF-β R1 and R2 primarily colocalize with MCs (42). In EoE, a disease similarly characterized by T2 inflammation and MC_T_ expansion, *TGFB1* mRNA and TGF-β targets within the epithelium are elevated (49, 50). We observed similar upregulation of *TGFB1* and *TGFB2* in basal EpC from CRSwNP relative to CRSsNP (Supplemental Figure 4). Interestingly, single nucleotide polymorphisms at or near the *TGFBR1*, *SMAD3*, and *SMAD6* loci are linked to asthma risk (51, 52), and at least 1 such polymorphism localizes to an open chromatin region in human MCs (32). Thus, elevated TGF-β signaling during T2 mucosal inflammation is closely linked with MC_T_ expansion, making it tempting to speculate that polymorphisms in the TGF-β signaling pathway could play a direct role in driving changes in MC function related to disease pathogenesis. While our findings support a likely role for TGF-β signaling in MC_T_ development across tissues and disease states, further studies will be required to confirm this hypothesis.

While PB-MC_TC_ treatment with TGF-β1 suppressed expression of *CMA1* and *CTSG* at the transcript level, it had minimal impact on intracellular protease content of either PB-MC_TC_s or primary MC_TC_s cultured ex vivo (Figure 3), suggesting that MC proteases are highly stable once packaged within secretory granules. However, both expression and function of MRGPRX2, an MC_TC_-associated G-protein–coupled receptor that plays a role in host defense and neuroimmune interactions, was suppressed following short-term TGF-β1 treatment (Figure 4). TGF-β1 treatment also enhanced PB-MC_TC_ eicosanoid production and modified cytokine, chemokine, and growth factor secretion following activation (Figure 5 and Figure 6). The contrasting stability of the MC_TC_ protease phenotype and plasticity of its surface and effector phenotype indicates the potential for the MC immunophenotype and effector phenotype to become uncoupled if TGF-β levels increase following MC maturation, adding an additional layer of complexity to MC heterogeneity. In contrast, we observed MC_T_ granule phenotype plasticity in both PB-MC_T_s and primary MC_T_s cultured ex vivo (Figure 3). This indicates that continued TGF-β signaling is required to maintain the MC_T_ phenotype and suggests that interruption in TGF-β signaling in the epithelium could lead to chymase incorporation, resulting in “ex-MC_T_” intraepithelial MCs with MC_TC_-like protease expression profiles. This has clear implications in diseases in which MC_TC_s can be observed in the epithelium, such as severe asthma and the glandular epithelium of nasal polyps (4, 6).

The differential expression of proinflammatory mediator transcripts between polyp MC_T_s and MC_TC_s (Figure 5A) suggested differential microenvironmentally regulated capacity to drive inflammation. Supporting this concept, PB-MC_T_s secreted more IL-5 but less IL-13 relative to PB-MC_TC_ following activation with either IL-33 or IgE crosslinking, mirroring the differential expression of these transcripts in polyp MCs (Figure 5). Both IL-5 and IL-13 are elevated in polyp sinonasal tissue extracts compared with controls, and IL-5 immunoreactivity was previously found to be restricted to MC_T_s in asthmatic bronchial mucosal biopsies (53, 54). This suggests enhanced MC_T_ crosstalk with cells expressing the IL-5 receptor, such as eosinophils and IL-5R–expressing plasma cells, both of which are notably elevated in CRSwNP (55–57). The reduction of polyp size observed following therapeutic IL-5 neutralization may work in part through countering the actions of IL-5 released by MC_T_s (58), whereas the efficacy of IL-4Rα blockade may in part reflect neutralization of MC_TC-_secreted IL-13 (59).

Our ex vivo studies indicated that MC_T_s are both transcriptionally and functionally primed for LTC_4_ and PGD_2_ generation relative to MC_TC_s and implicate TGF-β as a central driver of this distinction. This suggests MC_T_s may drive both the high steady-state levels of LTC_4_ and PGD_2_ metabolites identified in the urine of AERD patients and the subsequent increase in these mediators, in conjunction with elevations in serum histamine and tryptase, following clinical reactions to aspirin challenge (60, 61). CysLTs and PGD_2_ contribute to the severe T2 sinonasal and bronchial inflammation that characterizes AERD both through their actions as bronchoconstrictors and through eliciting chemotaxis and activation of T helper type 2 cells and group 2 innate lymphoid cells (62, 63). Notably, while our functional and transcriptional data indicate that TGF-β–inducible COX-1 is the dominant regulator of PGD_2_ production by PB-MC_T_s and PB-MC_TC_s in vitro, COX-2 is transcriptionally enriched in MC_TC_s in vivo. This may explain why no significant differences in PGD_2_ production were observed between primary MC subsets ex vivo and likely indicates the presence of other in vivo signals regulating MC eicosanoid production.

In summary, our data strongly support a role for TGF-β signaling within the epithelium inducing MC polarization toward the MC_T_ phenotype, thereby directly altering their effector capacity. TGF-β can drive MC_T_ differentiation in vitro, creating a powerful tool for investigating their role in diseases. However, PB-MC_T_ do not fully recapitulate the in vivo MC_T_ phenotype, underlining that the ultimate phenotype of inflammation-driven MC_T_s is likely directed by the combined effect of both microenvironment and inflammation-associated signals such as IL-4, previously shown to similarly upregulate a portion of nasal polyp MC_T_-associated transcripts (3). The selective potential of MC_T_s to produce T2-facilitating lipid mediators and IL-5 and MC_TC_s to produce IL-13 illustrate their discrete contribution to eosinophil-rich tissue inflammation and suggest the potential to therapeutically target individual MC subsets in instances where their principal activation products are major contributors to disease pathobiology.

## Methods

### Sex as a biological variable.

The study was conducted using samples from both female and male donors. Sex was not considered as a biological variable.

### Cell culture.

CD34^+^ cells were isolated from PB of human donors obtained from Brigham and Women’s Hospital. For RNA-Seq, buffy coats from consented donors were purchased from Research Blood Components, LLC. PB mononuclear cells were isolated using Ficoll paque density gradient centrifugation prior to CD34^+^ cell isolation using a CD34 MicroBead UltraPure Kit (Miltenyi Biotec) following the manufacturer’s protocol. Isolated cells were seeded at 150,000 cell/ml in StemSpan SFEM media (StemCell Technologies) containing SCF at 100 ng/ml, IL-6 at 50 ng/ml, IL-3 at 1 ng/ml for the first 3 weeks of culture (PeproTech), and penicillin/streptomycin at 1:100 (Invitrogen) (MC differentiation conditions). For studying the effect of TGF-β on MC development, TGF-β1 (PeproTech) at 1 ng/ml was added to CD34^+^ cells cultured under MC differentiation conditions as defined above and maintained for the duration of culture. Media was changed weekly, and after 7 weeks, the cells had reached at least 95% purity and were used for downstream experiments. For short-term TGF-β1 treatment, mature MCs were treated for 6 days with 1 ng/ml of TGF-β1 in StemSpan media (STEMCELL Technologies) containing SCF at 100 ng/ml.

For ex vivo culture, MC_T_s and MC_TC_s were flow sorted from dissociated nasal polyp tissue and cultured for 2 weeks in StemSpan SFEM media containing 100 ng/ml SCF with or without 1 ng/ml TGF-β1.

For MC/EpC coculture, PB-MC_T_s were seeded with primary sinus EpCs expanded ex vivo from ethmoid sinus tissue as previously described (13) and used at passage 1 or 2. MCs and EpCs were seeded at a 1:5 ratio (MC:EpC) on trans-well inserts (Corning) coated with collagen (STEMCELL Technologies) for 9 days with media changes every 3 days. Coculture media consisted of a 50% (v/v) mixture of StemSpan SFEM and PneumaCult Ex (STEMCELL Technologies), supplemented with SCF at 100 ng/ml and IL-6 at 50 ng/ml, and maintained for the duration of coculture in the presence or absence of the TGF-βR dual kinase inhibitor LY2109761 (Cayman) at defined concentrations.

### Cell activation.

PB-MCs were incubated with human myeloma IgE (Athens Research and Technology) at 2 μg/ml overnight and washed prior to activation with anti-human IgE (Chemicon) at 5 μg/ml in cell culture media containing 100 ng/ml SCF. In other experiments, cells were activated with IL-33 (PeproTech) at 10 ng/ml or the MRGPRX2 ligands substance P (MilliporeSigma) and compound 48/80 (MilliporeSigma) as described in figure legends. Culture supernatants were collected following stimulation for ELISA analysis. For flow cytometric evaluation of MC degranulation, anti-human LAMP-1 was added to the samples at the time of cell activation. One hour after activation, cells were collected and washed followed by flow cytometric evaluation of cell-surface LAMP-1.

To activate nasal polyp MC subsets ex vivo, flow-sorted cells were rested overnight in StemSpan SFEM containing 100 ng/ml SCF. Cells were activated with anti-human IgE (Chemicon) at 5 μg/ml in StemSpan SFEM containing 100 ng/ml SCF for 1 hour.

For additional Methods, see Supplemental Methods.

### Statistics.

Number of biological samples included in analyses are listed throughout figure legends. Statistical analyses were performed using GraphPad Prism, version 8.1.2, and DESeq2 or Seurat 5, implemented in RStudio. Violin plots were generated using Graphpad Prism using data exported from Seurat to allow display of median and quartile values. For comparison of 2 samples, the ratio paired *t* test or Mann-Whitney *U* test (2-tailed) was used where appropriate. For comparison of 3 or more samples, 1-way ANOVA of transformed values (natural logarithm) or nonparametric test was used where appropriate. For RNA-Seq analysis, FDR values of less than 0.05 were considered significant, with an additional cutoff of log_2_FoldChange greater than 0.5 where indicated. For all nonsequencing-based experiments, *P* < 0.05 (following adjustment for multiple comparisons when applicable) was considered significant.

### Study approval.

Ethmoid sinus tissue was obtained from participants between the ages of 20 to 80 years recruited from the Brigham and Women’s Hospital Allergy and Immunology clinics and Otolaryngology clinics between 2019 and 2022 (Supplemental Table 7). The study was approved by the Institutional Review Board with all study participants providing written consent. Tissue was collected at the time of elective endoscopic sinus surgery from patients with physician-diagnosed CRSwNP or CRSsNP. Patients with polyps included diagnosis of both aspirin-tolerant CRSwNP and aspirin-intolerant AERD. A diagnosis of AERD was suspected for patients with asthma, nasal polyposis, and a history of respiratory reaction following usage of cyclooxygenase inhibitors, and all diagnoses were confirmed via graded oral challenge with aspirin.

### Data availability.

Underlying data for all figures can be found in the associated raw data file. Full differential expression analysis and pathway analysis can be found in supplemental data. Sequencing data for single-cell RNA-Seq and bulk RNA-Seq have been deposited in the NCBI’s Gene Expression Omnibus (GEO GSE279289 and GSE279240, respectively). Values for all data points in graphs are reported in the Supporting Data Values file.

## Author contributions

TD and DFD conceived the study and designed experiments. KMB and TML oversaw and coordinated human sample collection. TR and AZM contacted patients for consent and collected samples and patient information. JH processed tissue samples and maintained a patient sample biobank. Sinus surgeries were conducted by RWB, NB, SEL, AM, and RER. TD and EH processed tissue samples and conducted all experiments. AP and LB aided in tissue staining and microscopic data collection and analysis. TD and DFD analyzed data and interpreted results. TD, JAB, and DFD wrote the manuscript, with input from all authors.

## Supplementary Material

Supplemental data

Supplemental table 1

Supplemental table 2

Supplemental table 3

Supplemental table 4

Supplemental table 5

Supplemental table 6

Supplemental table 7

Supporting data values

## Version 1 01/02/2025

Electronic publication

## References

[1] Gurish MF, Austen KF (2012). Developmental origin and functional specialization of mast cell subsets. Immunity.

[2] Metcalfe DD (1997). Mast cells. Physiol Rev.

[3] Dwyer DF (2021). Human airway mast cells proliferate and acquire distinct inflammation-driven phenotypes during type 2 inflammation. Sci Immunol.

[4] Takabayashi T (2012). Glandular mast cells with distinct phenotype are highly elevated in chronic rhinosinusitis with nasal polyps. J Allergy Clin Immunol.

[5] Abonia JP (2010). Involvement of mast cells in eosinophilic esophagitis. J Allergy Clin Immunol.

[6] Balzar S (2011). Mast cell phenotype, location, and activation in severe asthma. Data from the Severe Asthma Research Program. Am J Respir Crit Care Med.

[7] Bentley AM (1992). Immunohistology of the nasal mucosa in seasonal allergic rhinitis: increases in activated eosinophils and epithelial mast cells. J Allergy Clin Immunol.

[8] Dougherty RH (2010). Accumulation of intraepithelial mast cells with a unique protease phenotype in T(H)2-high asthma. J Allergy Clin Immunol.

[9] James A (2012). Corticosteroid treatment selectively decreases mast cells in the smooth muscle and epithelium of asthmatic bronchi. Allergy.

[10] Altman MC (2019). Airway epithelium-shifted mast cell infiltration regulates asthmatic inflammation via IL-33 signaling. J Clin Invest.

[11] Derakhshan T (2020). Lineage-specific regulation of inducible and constitutive mast cells in allergic airway inflammation. J Exp Med.

[12] Korsunsky I (2019). Fast, sensitive and accurate integration of single-cell data with Harmony. Nat Methods.

[13] Ordovas-Montanes J (2018). Allergic inflammatory memory in human respiratory epithelial progenitor cells. Nature.

[14] Olsson N (2000). Human mast cell migration in response to members of the transforming growth factor-beta family. J Leukoc Biol.

[15] Kubiczkova L (2012). TGF-β - an excellent servant but a bad master. J Transl Med.

[16] Sugimoto K (2012). The αvβ6 integrin modulates airway hyperresponsiveness in mice by regulating intraepithelial mast cells. J Clin Invest.

[17] Travis MA, Sheppard D (2014). TGF-β activation and function in immunity. Annu Rev Immunol.

[18] Alladina J (2023). A human model of asthma exacerbation reveals transcriptional programs and cell circuits specific to allergic asthma. Sci Immunol.

[19] Smillie CS (2019). Intra- and inter-cellular rewiring of the human colon during ulcerative colitis. Cell.

[20] Rönnberg E (2012). Mast cell proteoglycans. J Histochem Cytochem.

[21] Forsberg E (1999). Abnormal mast cells in mice deficient in a heparin-synthesizing enzyme. Nature.

[22] Matsuzawa S (2003). IL-9 enhances the growth of human mast cell progenitors under stimulation with stem cell factor. J Immunol.

[23] Coffey CS (2009). Mucosal expression of nerve growth factor and brain-derived neurotrophic factor in chronic rhinosinusitis. Am J Rhinol Allergy.

[24] Olgart Höglund C (2002). Nerve growth factor levels and localisation in human asthmatic bronchi. Eur Respir J.

[25] Lorentz A (2005). IL-4-induced priming of human intestinal mast cells for enhanced survival and Th2 cytokine generation is reversible and associated with increased activity of ERK1/2 and c-Fos. J Immunol.

[26] Kajiwara N (2010). Activation of human mast cells through the platelet-activating factor receptor. J Allergy Clin Immunol.

[27] Gaudenzio N (2016). Different activation signals induce distinct mast cell degranulation strategies. J Clin Invest.

[28] Woolhiser MR (2004). Activation of human mast cells by aggregated IgG through FcgammaRI: additive effects of C3a. Clin Immunol.

[29] Gomez G (2005). TGF-beta 1 inhibits mast cell Fc epsilon RI expression. J Immunol.

[30] Ndaw VS (2017). TGF-β1 Suppresses IL-33-induced mast cell function. J Immunol.

[31] Kumar M (2021). Unlocking the non-IgE-mediated pseudo-allergic reaction puzzle with Mas-related G-protein coupled receptor member X2 (MRGPRX2). Cells.

[32] Cildir G (2019). Genome-wide analyses of chromatin state in human mast cells reveal molecular drivers and mediators of allergic and inflammatory diseases. Immunity.

[33] Desai A (2016). IL-6 promotes an increase in human mast cell numbers and reactivity through suppression of suppressor of cytokine signaling 3. J Allergy Clin Immunol.

[34] Toru H (1998). Interleukin-4 promotes the development of tryptase and chymase double-positive human mast cells accompanied by cell maturation. Blood.

[35] Hsieh FH (2005). Human airway epithelial cell determinants of survival and functional phenotype for primary human mast cells. Proc Natl Acad Sci U S A.

[36] Ojiaku CA (2017). Transforming growth factor β1 function in airway remodeling and hyperresponsiveness. The missing link?. Am J Respir Cell Mol Biol.

[37] Halwani R (2011). Role of transforming growth factor-β in airway remodeling in asthma. Am J Respir Cell Mol Biol.

[38] Denney L (2015). Pulmonary epithelial cell-derived cytokine TGF-β1 is a critical cofactor for enhanced innate lymphoid cell function. Immunity.

[39] Wahl SM (1987). Transforming growth factor type beta induces monocyte chemotaxis and growth factor production. Proc Natl Acad Sci U S A.

[40] Wipff PJ, Hinz B (2008). Integrins and the activation of latent transforming growth factor beta1 - an intimate relationship. Eur J Cell Biol.

[41] Puddicombe SM (2000). Involvement of the epidermal growth factor receptor in epithelial repair in asthma. FASEB J.

[42] Howat WJ (2002). TGF-beta isoform release and activation during in vitro bronchial epithelial wound repair. Am J Physiol Lung Cell Mol Physiol.

[43] Breuss JM (1995). Expression of the beta 6 integrin subunit in development, neoplasia and tissue repair suggests a role in epithelial remodeling. J Cell Sci.

[44] Torrego A (2007). Expression and activation of TGF-beta isoforms in acute allergen-induced remodelling in asthma. Thorax.

[45] Batra V (2004). Bronchoalveolar lavage fluid concentrations of transforming growth factor (TGF)-beta1, TGF-beta2, interleukin (IL)-4 and IL-13 after segmental allergen challenge and their effects on alpha-smooth muscle actin and collagen III synthesis by primary human lung fibroblasts. Clin Exp Allergy.

[46] Redington AE (1997). Transforming growth factor-beta 1 in asthma. Measurement in bronchoalveolar lavage fluid. Am J Respir Crit Care Med.

[47] Salib RJ (2004). Nasal mucosal immunoexpression of the mast cell chemoattractants TGF-beta, eotaxin, and stem cell factor and their receptors in allergic rhinitis. J Allergy Clin Immunol.

[48] de Boer WI (1998). Transforming growth factor beta1 and recruitment of macrophages and mast cells in airways in chronic obstructive pulmonary disease. Am J Respir Crit Care Med.

[49] Mishra A (2008). Esophageal remodeling develops as a consequence of tissue specific IL-5-induced eosinophilia. Gastroenterology.

[50] Aceves SS (2007). Esophageal remodeling in pediatric eosinophilic esophagitis. J Allergy Clin Immunol.

[51] Olafsdottir TA (2020). Eighty-eight variants highlight the role of T cell regulation and airway remodeling in asthma pathogenesis. Nat Commun.

[52] Demenais F (2018). Multiancestry association study identifies new asthma risk loci that colocalize with immune-cell enhancer marks. Nat Genet.

[53] Stevens WW (2015). Cytokines in chronic rhinosinusitis. Role in eosinophilia and aspirin-exacerbated respiratory disease. Am J Respir Crit Care Med.

[54] Bradding P (1995). Heterogeneity of human mast cells based on cytokine content. J Immunol.

[55] Buchheit KM (2020). IL-5Rα marks nasal polyp IgG4- and IgE-expressing cells in aspirin-exacerbated respiratory disease. J Allergy Clin Immunol.

[56] Jankowski R (2002). Clinical factors influencing the eosinophil infiltration of nasal polyps. Rhinology.

[57] Al-Shaikhly T (2021). Location of eosinophils in the airway wall is critical for specific features of airway hyperresponsiveness and T2 inflammation in asthma. Eur Respir J.

[58] Gevaert P (2011). Mepolizumab, a humanized anti-IL-5 mAb, as a treatment option for severe nasal polyposis. J Allergy Clin Immunol.

[59] Bachert C (2019). Efficacy and safety of dupilumab in patients with severe chronic rhinosinusitis with nasal polyps (LIBERTY NP SINUS-24 and LIBERTY NP SINUS-52): results from two multicentre, randomised, double-blind, placebo-controlled, parallel-group phase 3 trials. Lancet.

[60] Fischer AR (1994). Direct evidence for a role of the mast cell in the nasal response to aspirin in aspirin-sensitive asthma. J Allergy Clin Immunol.

[61] Bochenek G (2003). A controlled study of 9alpha,11beta-PGF2 (a prostaglandin D2 metabolite) in plasma and urine of patients with bronchial asthma and healthy controls after aspirin challenge. J Allergy Clin Immunol.

[62] Salimi M (2017). Cysteinyl leukotriene E_4_ activates human group 2 innate lymphoid cells and enhances the effect of prostaglandin D_2_ and epithelial cytokines. J Allergy Clin Immunol.

[63] Pettipher R (2007). Antagonism of the prostaglandin D2 receptors DP1 and CRTH2 as an approach to treat allergic diseases. Nat Rev Drug Discov.

