# Supplementary material for: Human intraepithelial mast cell differentiation and effector function are directed by TGF-β signaling
Source: J Clin Invest. 2025 Jan 2;135(1):e174981. doi: 10.1172/JCI174981 (PMC11684804; doi:10.1172/JCI174981)
Supplement: Supplemental data [file jci-135-174981-s189.pdf]

Supplementary Methods

Antibodies

The following anti-human antibodies were purchased from BioLegend: CD117 (clone 104D2), FcεR1a (clone CRA-1), MRGPRX2 (clone K125H4), CD45 (clone 2D1), CD11b (clone ICRF44), CD11c (clone 3.9), CD38 (clone HB-7), CD3 (clone OKT3), CD19 (clone HIB193), LAMP-1 (clone H4A3), CD31 (clone WM59), CD90 (clone 5E10), EpCAM (clone 9C4), NGFR (clone ME20.4), TGF-βR2 (clone W17055E), and mouse IgG1 isotype control (clone MOPC-21). Anti-human CPA3 (clone CA2) was from Immunostep S.L, anti-human chymase antibody (clone B7) and anti-human αVβ6 (clone E7P6) were from Chemicon, and anti-human CTSG (clone AHN-11) was from Millipore Sigma. Phospho-SMAD2/SMAD3 polyclonal antibody was purchased from Invitrogen as well as donkey anti-rabbit IgG (H+L) and goat anti-mouse IgG (H+L) secondary antibodies.

Flow cytometry

To purify MC subsets, MCs were flow sorted on a Sony SH800S cell sorter using Sony Cell Sorter Software from single cell suspensions obtained from dissociation of ethmoid sinus using a MC surface marker panel ($CD45^{+}$, $CD117^{+}$, $Fc\varepsilon R1\alpha^{+}$, $CD11b^{-}$, $CD11c^{-}$, $CD3^{-}$, $CD19^{-}$) and divided into MC_{TC} and MC_{T}s based on differential expression of CD38 and CD117, based on previously published criteria (3).

PB-MCs or disassociated ethmoid sinus cells were incubated with human Truestain FcX (BioLegend) prior to staining to block antibody non-specific binding. Cell surface markers were stained using the antibody cocktail for 40 min on ice. For detection of the intracellular markers (CPA3, chymase, and CTSG), cells were fixed and permeabilized using BD Cytofix/Cytoperm fixation/permeabilization solution kit (BD bioscience) following the manufacturer's protocol. Cells were incubated in blocking buffer containing 1% BSA and human Truestain FcX for at least 15 min prior to staining for intracellular markers for 30 min at room temperature. Anti-human Chymase antibody and anti-human CTSG were conjugated to AF647 and AF488 fluorophores, respectively, following the manufacturer's protocol (Invitrogen). For integrin αVβ6 expression evaluation, the cells were stained with anti-human αVβ6 or isotype control prior to staining with the secondary antibody AF647

and cell surface markers for 45 min on ice. Cell viability was measured by staining with a viability staining solution (Zombie fixable viability dye or 7-AAD, BioLegend). For conventional flow cytometry, cells were collected on a BD CANTO-II using BD FACSDiva software.

RNA-sequencing sample preparation and analysis

For single cell RNA sequencing, MCs from the nasal polyps of four aspirin-intolerant donors were used. Single cell suspensions were obtained from dissociation of surgical specimens from ethmoid sinus as previously described (3). Briefly, the tissue was minced and digested in a solution containing 600 U/ml collagenase IV (Worthington), 25 μg/ml DNase (Roche), and 10% fetal bovine serum (FBS). Red blood cells were lysed prior to staining the single cell suspensions with MC surface marker panel ($CD45^+$, $CD117^+$, $Fc\varepsilon R1\alpha^+$, $CD11b^-$, $CD11c^-$, $CD3^-$, $CD19^-$) to flow sort all MCs expressing CD117 and $Fc\varepsilon R1\alpha$. Dead cells were excluded using propidium iodide or Zombie viability dye (BioLegend). Sorted cells were resuspended in 0.4% BSA in PBS at a concentration of 1,000 cells per µl, and loaded onto a single lane (Chromium chip, 10X Genomics) for encapsulation in lipid droplets, using the Single Cell 3′kit V3.1 (10X Genomics). cDNA synthesis, gene expression and protein library generation were performed according to the 10X Genomics protocol. The generated gene expression libraries were sequenced to an average of 30,000 reads per cell, and the surface protein libraries to an average of 15,000 reads per cell, using the Illumina Novaseq platform. ScRNA-seq reads were processed with Cell Ranger, which quantified transcript counts per putative cell. Quantification was performed using the STAR aligner against the GRCh38 transcriptome.

The filtered matrices were individually imported into Seurat v5 (66). The scDblFinder package was used to identify likely doublets. For each sample, a dynamic upper limit for detected genes and unique molecular identifiers was set at the 75th percentile + 1.5* interquartile range on a per-sample basis, and a lower limit of 350 genes and 600 unique molecular identifiers was used, with all cells not meeting these criteria filtered out. Cells with greater than 20% mitochondrial genes or an scDblFinder score > 0.5 were excluded, after which matrices were merged prior to downstream analysis. The

total number of cells passing these filters captured across all patients was 22,722, for an average of 5,681 cells per donor. Before performing dimensionality reduction, data was normalized and scaled using the scTransform method. Data from the four donors was integrated using the Harmony package for R (version 1.2) using default settings (12). A shared nearest neighbor (SNN) graph and uniform manifold approximation and projection (UMAP) embedding were constructed using the first 50 harmony components. The FindClusters function was used with the default resolution of 0.8 and RunUMAP used with the default settings to identify 20 clusters across the four input samples. The seven clusters containing MC genes consistently observed across all donors without other contaminating populations were computationally subsetted, after which scTransform was run a second time, principal component analysis conducted, and data re-integrating using Harmony with the same settings as before. The first 18 harmony components were chosen used to construct a SNN graph and UMAP embeddings following visual inspection of the elbow plot, and clusters were determined at a resolution of 0.4 based on stability after evaluating all resolutions between 0.1 and 1 using the clustree package for R (67). Quality control plots and violin plots were generated using the scCustomize package for R (68).

Differential gene expression analysis between clusters was conducted using a pseudobulk approach, based on recent benchmarking studies (69). Raw counts within each cluster were aggregated by donor, after which differential gene expression analysis was conducted using DESeq2, contrasting each cluster to the average of all other clusters. All transcripts with fewer than 2,500 total counts across all replicates were removed from analysis. Transcripts identified as differentially expressed between clusters with an adjusted p value of less than 0.05 were considered statistically significant.

For re-analysis of existing human nasal polyp MCs and human colon datasets (3, 19), datasets were obtained from dbGaP (phs002333.v1.p1) or the Broad single-cell portal (SCP259), respectively. MCs were selected based on cluster designations assigned in each study. For re-analysis of human sinus and lung epithelium, datasets were obtained from the Broad single-cell portal (SCP253) or the gene expression omnibus (GSE193816) (13, 18).

For bulk RNA sequencing of PB-MCs, MCs from three donors grown under standard conditions were treated with or without 1 ng/ml TGF-β1 for 24 hours or six days. In separate experiments, MCs from three separate donors were grown for seven weeks under standard conditions in the presence or absence of 1 ng/ml TGF-β1. Cells were collected in TCL buffer with 1% 2-mercaptoethanol, and technical replicates (two per sample) were sequenced through the Broad Institute Genomics Platform (Boston, MA). SmartSeq2 libraries were prepared according to the SmartSeq2 protocol described by Trombetta et al, 2014 (70). Briefly, total RNA was purified using RNA-SPRI beads. cDNA was generated from full-length mRNA transcripts using reverse transcriptase with terminal transferase activity. Combined with a second "template switch" primer, the cDNA was constructed to have two universal priming sequences. Following preamplification, the Nextera XT library construction kit was used to prepare 96 unique indexes specific to each sample. Barcoded cDNA fragments were then pooled prior to sequencing. Sequencing was carried out using an Illumina NextSeq500 as paired- end 2x38bp to a coverage of approximately ~4 million reads per well. Reads were pseudoaligned to the human GrCH38 assembly using Kallisto (71) with default settings imported into R using TXImport, and concatenated and analyzed using DESeq2 (72, 73). Transcripts identified as differentially expressed between clusters (scRNA-seq) or replicates (bulk RNAseq) with a false discovery rate (FDR) of less than 0.05 were considered statistically significant.

Gene signatures of TGF-β upregulated transcripts were constructed using the top uniquely upregulated transcripts (up to 200) from each timepoint (defined as Log2FC > 0.5 and FDR<0.05) rank-ordered by FDR. Signatures were then mapped onto scRNA-seq datasets as percentage of total transcripts on a per-cell basis or used for gene set enrichment analysis. Genes associated with growth factors, cytokines, and transcription factors were incorporated from gene ontology annotations for growth factor activity (GO: 0008083), KEGG cytokine-cytokine receptor interaction hsa04060 pathway, and Lamber et al. (2018), respectively (74). Data was visualized using the built-in Seurat visualization options, the pheatmap and ggplot2 packages for R (75, 76).

Tissue staining and microscopy

Tissue sections were fixed with 50% (v/v) acetone in methanol prior to blocking with 3% donkey serum at room temperature. Tissue sections were incubated with phospho-SMAD2/SMAD3 antibody (1:200 dilution) in blocking solution overnight at 4C. After rinses, sections were incubated with donkey anti-rabbit AF546 secondary antibody (1:500 dilution) and DAPI for nuclear staining for one h at room temperature. The slides were coverslipped using a mounting media for microscopy.

ELISA

Supernatants from stimulated MC samples were stored at -80C and used for assessing various mediators. PGD_2 and CysLT ELISAs were purchased from Cayman. For measurement of protein mediator secretion, the flow cytometry-based Legendplex multiplex ELISA assay system (BioLegend) was used. For tryptase β2 and CPA3 release, ELISAs were purchased from Invitrogen and antibodies-online.com, respectively.

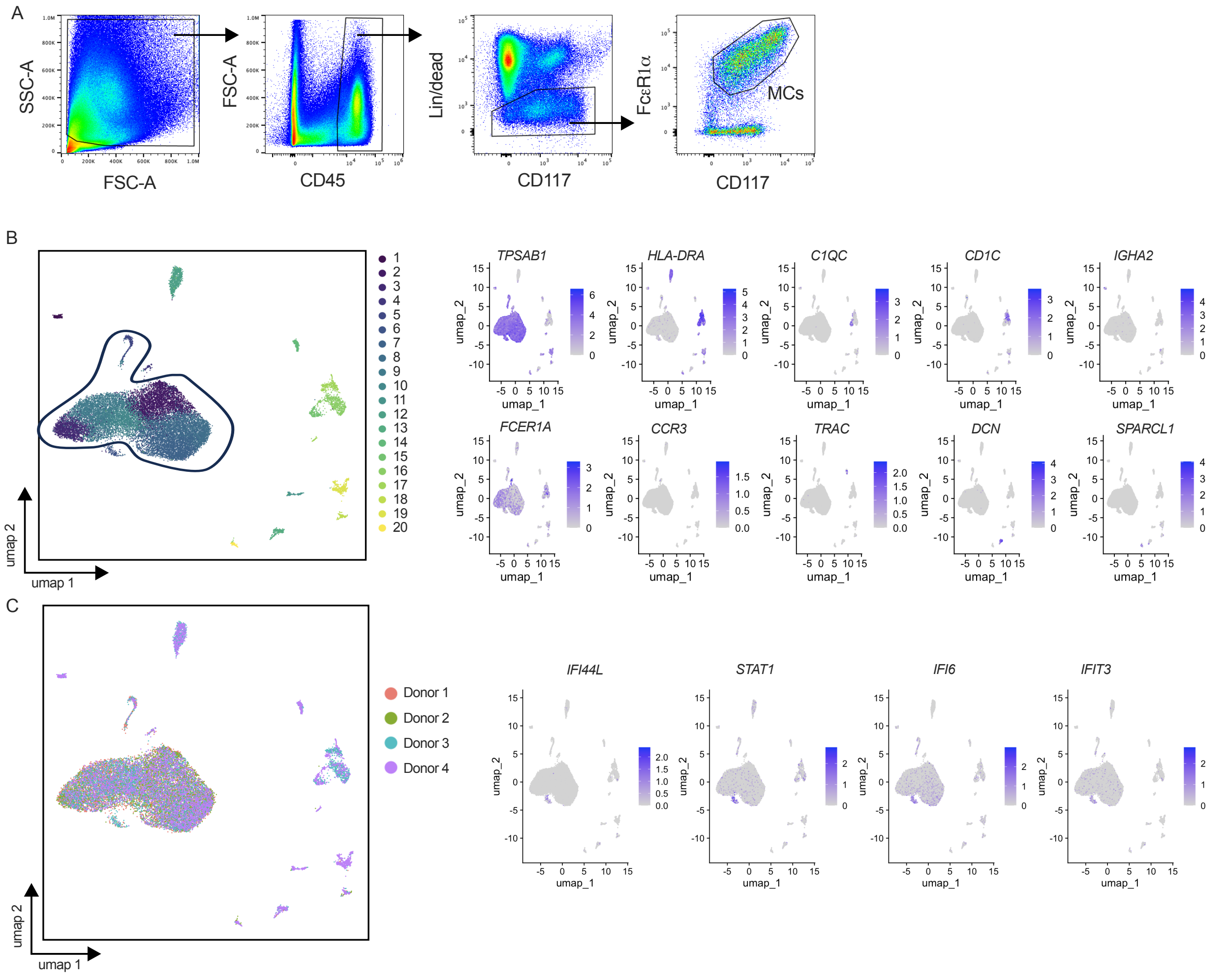

Fig. S1. scRNA-seq characterization of nasal polyp MCs. (**A**) Representative flow plots of gating strategy to identify and sort MCs from nasal polyps for scRNA-seq. (**B**) Uniform Manifold Approximation and Projection (UMAP) depiction of MCs and contaminating populations sorted from nasal polyps and expression pattern of *TPSAB1* and *CPA3* transcripts distinguishing MCs from contaminating myeloid (*HLA-DRA*, *C1QC*, *CD1C*), plasma cells (*IGHA2*), eosinophil/basophil (*CCR3*), lymphoid (*TRAC*), fibroblasts (*DCN*), and endothelial (*SPARCL1*) populations. (**C**) Identification of a donor-specific cluster enriched for interferon response genes that was excluded from downstream analysis.

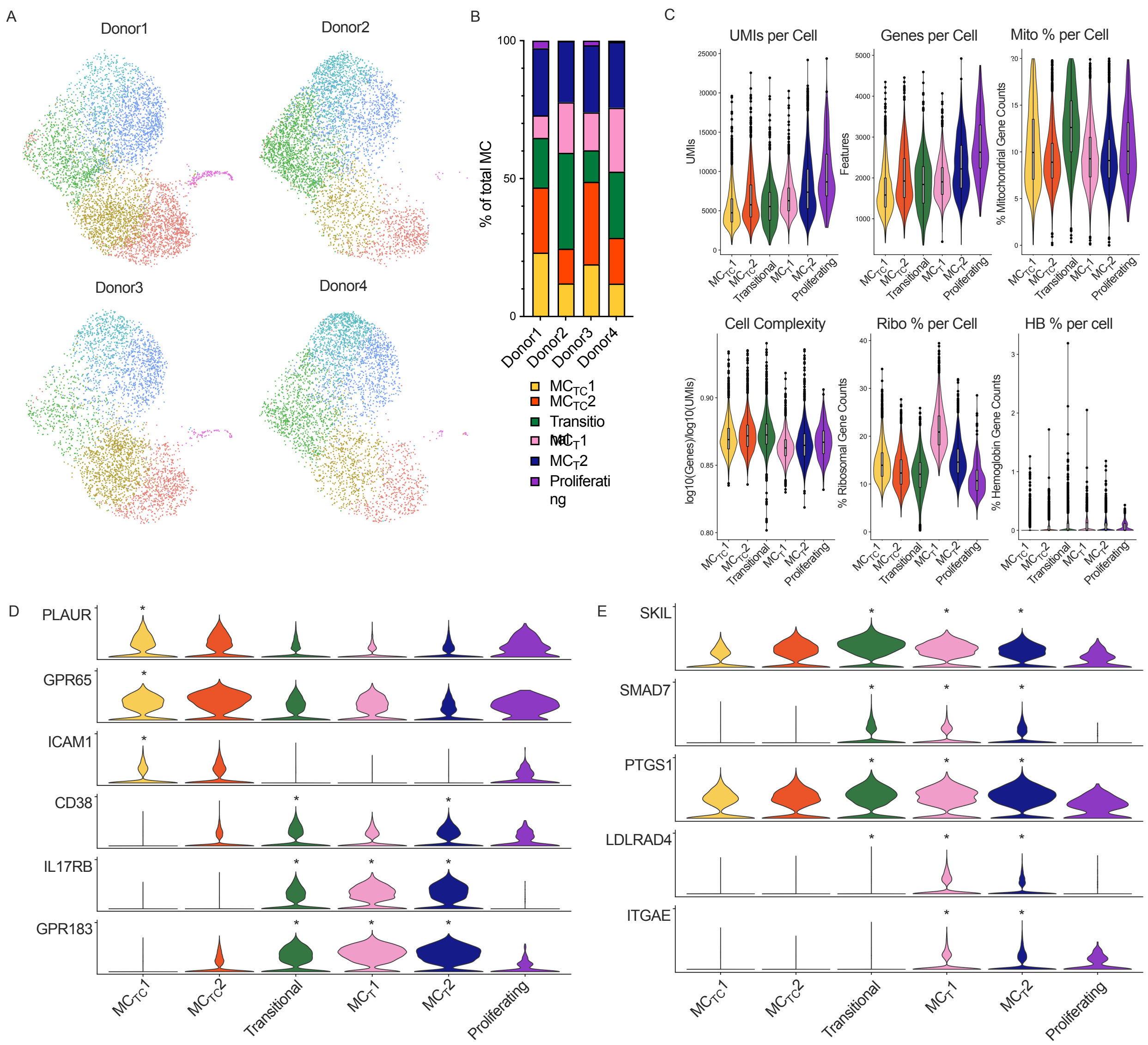

Fig. S2. QC analysis of nasal polyp MCs. (**A**) Display of identified clusters on a per-donor basis and (**B**) quantification of each cluster as a percentage of total cells per donor. (**C**) Violin plots showing total UMI per cell, genes per cell, complexity, mitochondrial percentage, ribosomal percentage, and hemoglobin gene percentage divided by clyster. (**D**) Violin plots showing select MC_T and MC_{TC}-associated transcripts differentially expressed across clusters. (**E**) Violin plots showing expression of murine MC TGF-β target genes differentially expressed across clusters. * indicates FDR < 0.05 (DESeq2).

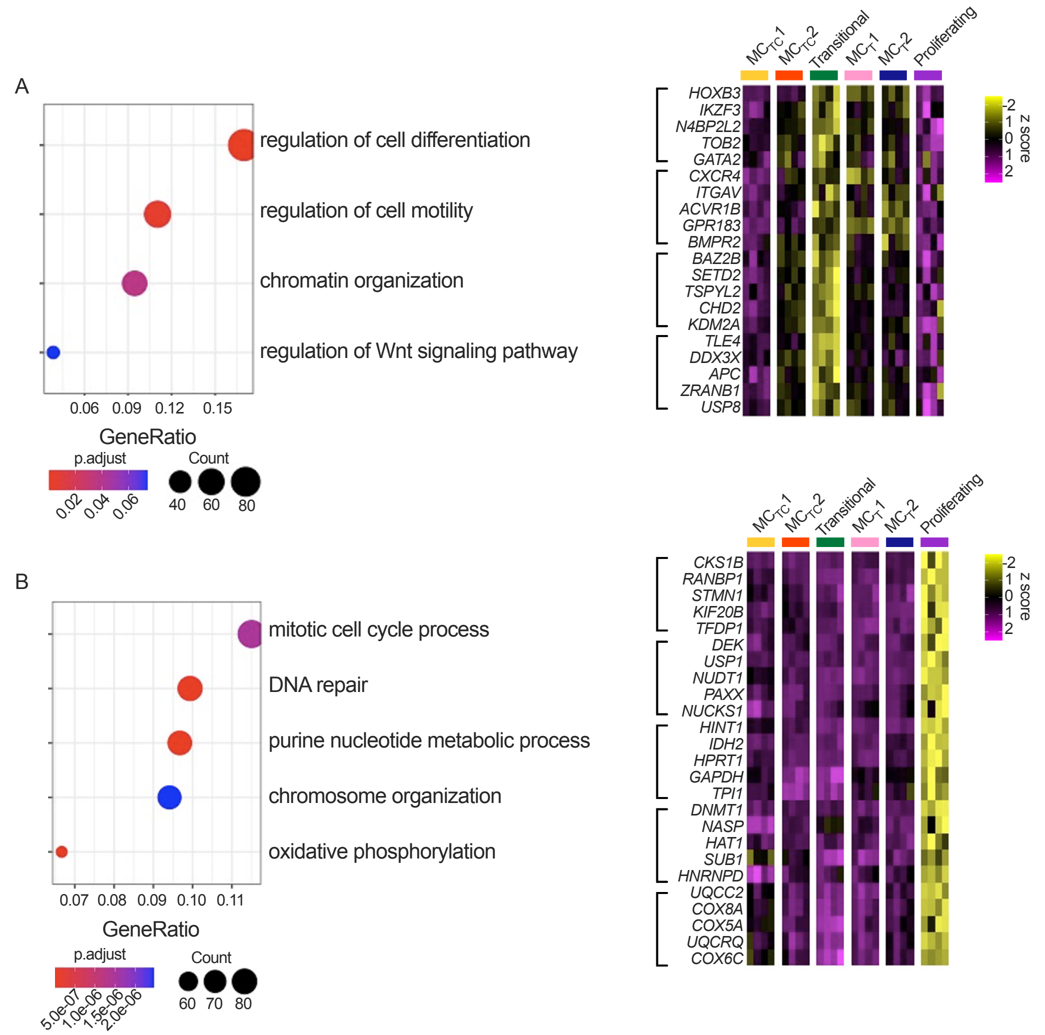

Fig. S3. **Gene enrichment analysis of proliferating and transitional clusters in nasal polyps.** Enrichment of biological processes in (**A**) transitional and (**B**) proliferating clusters in comparison with other clusters and row-normalized heatmaps showing representative expression of genes associated with the biological processes. Heatmap columns indicate average cluster expression for each of n= 4 individuals, FDR< 0.05 (DESeq2) and Log2 Fold Change> 0.5. Scale bars denote z score.

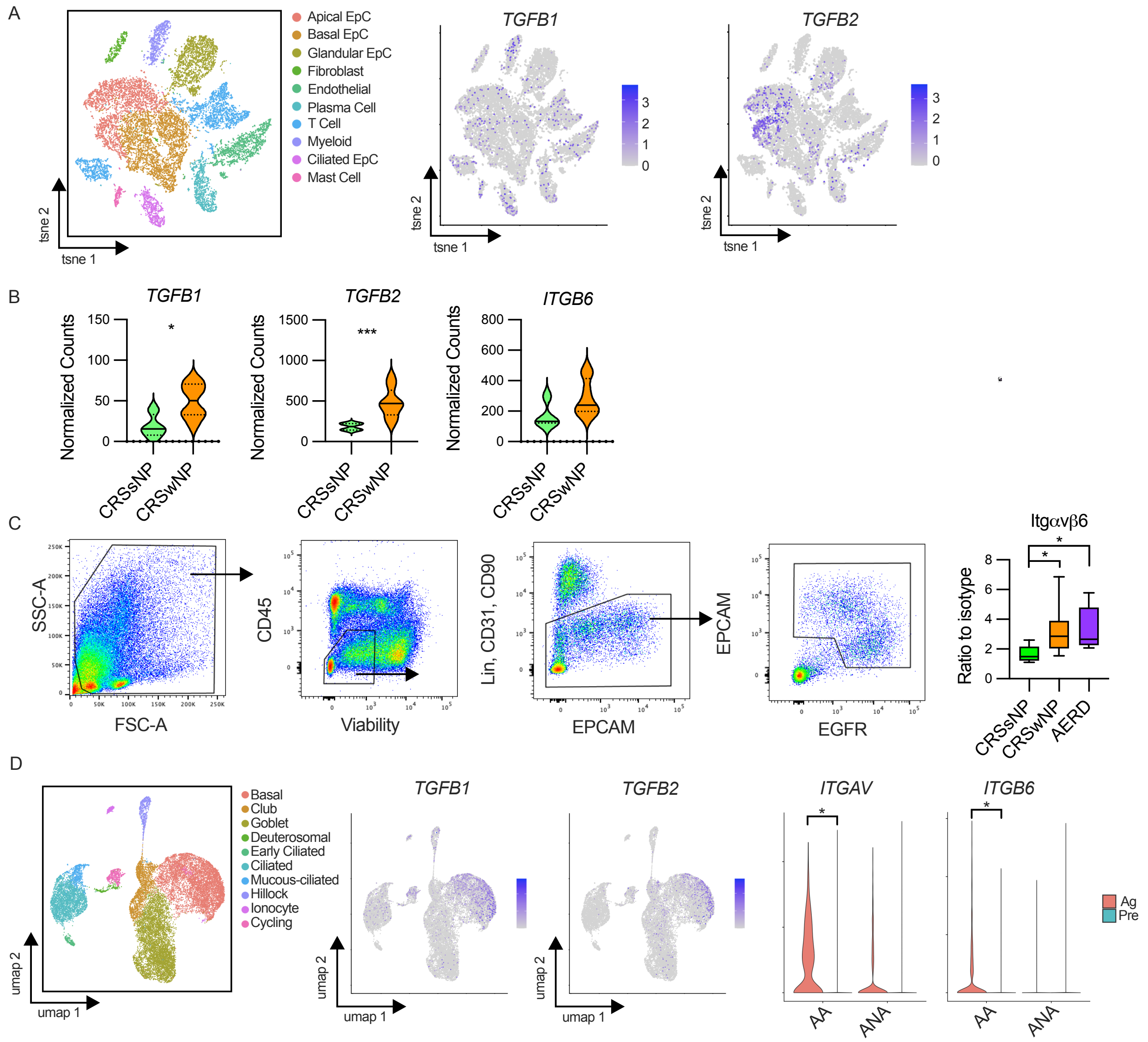

Fig. S4. Association between epithelium and TGF-β. (**A**) UMAP depiction of identified populations in human nasal polyps and expression patterns of *TGFB1* and *TGFB2* transcripts across populations. (**B**) Enrichment in *TGFB1*, *TGFB2*, and *ITGB6* transcripts in basal epithelial cells from polyp versus non-polyp tissue. Data from n = 8 and 4 individual donors, respectively. * padj < 0.05 and *** padj < 0.001 (DESeq2). (**C**) Flow gating scheme to identify sinus epithelial cells (left), with quantification of αVβ6 expression in epithelial cells across disease states (right). n= 5, 11, 9 individual donors for CRSsNP, CRSwNP, and AERD, respectively, * padj < 0.05 (ANOVA). (**D**) UMAP depiction of epithelial cells found in the lungs and expression pattern of *TGFB1* and *TGFB2* transcripts across epithelial cell phenotypes (left) and violin plots depicting enrichment of *ITGAV* and *ITGB6* in the lung basal epithelial cells across allergic asthmatics (AA) and allergic non-asthmatic (ANA) patients pre and post challenge with an allergen (Ag) (right). * FDR< 0.05 (DESeq2). Data from n = 4 individual donors per group. Box-and-whisker plots show median, interquartile range, and minimum/maximum values observed.

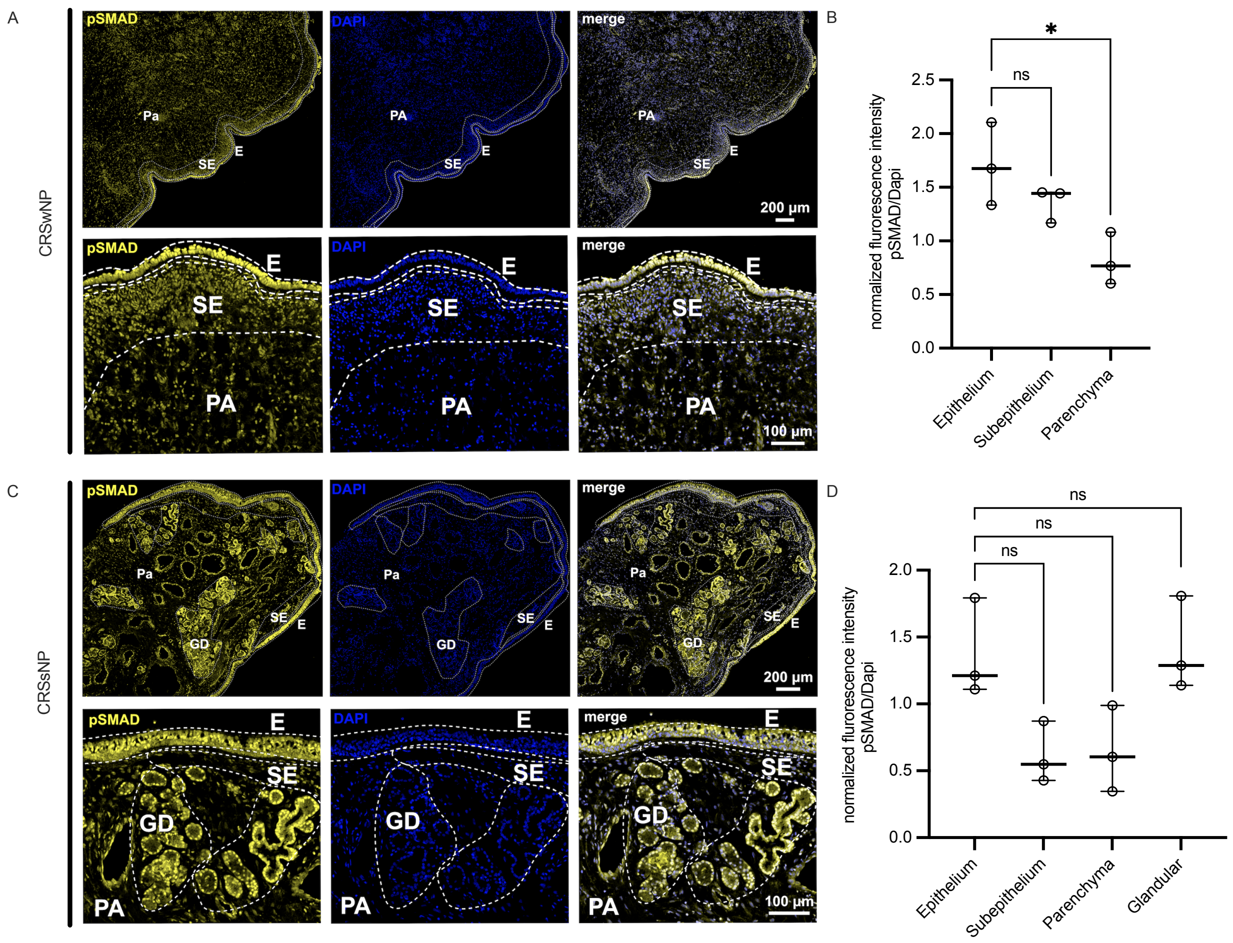

Fig. S5. SMAD3 phosphorylation in human sinus tissue. (**A**) Representative low magnification (top row) and high magnification (bottom row) images showing pSMAD staining (yellow) and DAPI (blue), together with merged images and masks for defining epithelial region (E), subepithelial region (SE) and parenchymal region (PA) for CRSwNP. (**B**) Quantification of pSMAD3, normalized to pSMAD3 to correct for differences in cell numbers, across the indicated regions for n=3 CRSwNP donors, * padj< 0.05, ns not significant (ANOVA). (**C**) Representative regions for CRSsNP, additionally showing masks for defining glandular (GD) region. (**D**) Normalized quantification of pSMAD3 across regions for n=3 CRSsNP donors, ns not significant (ANOVA).

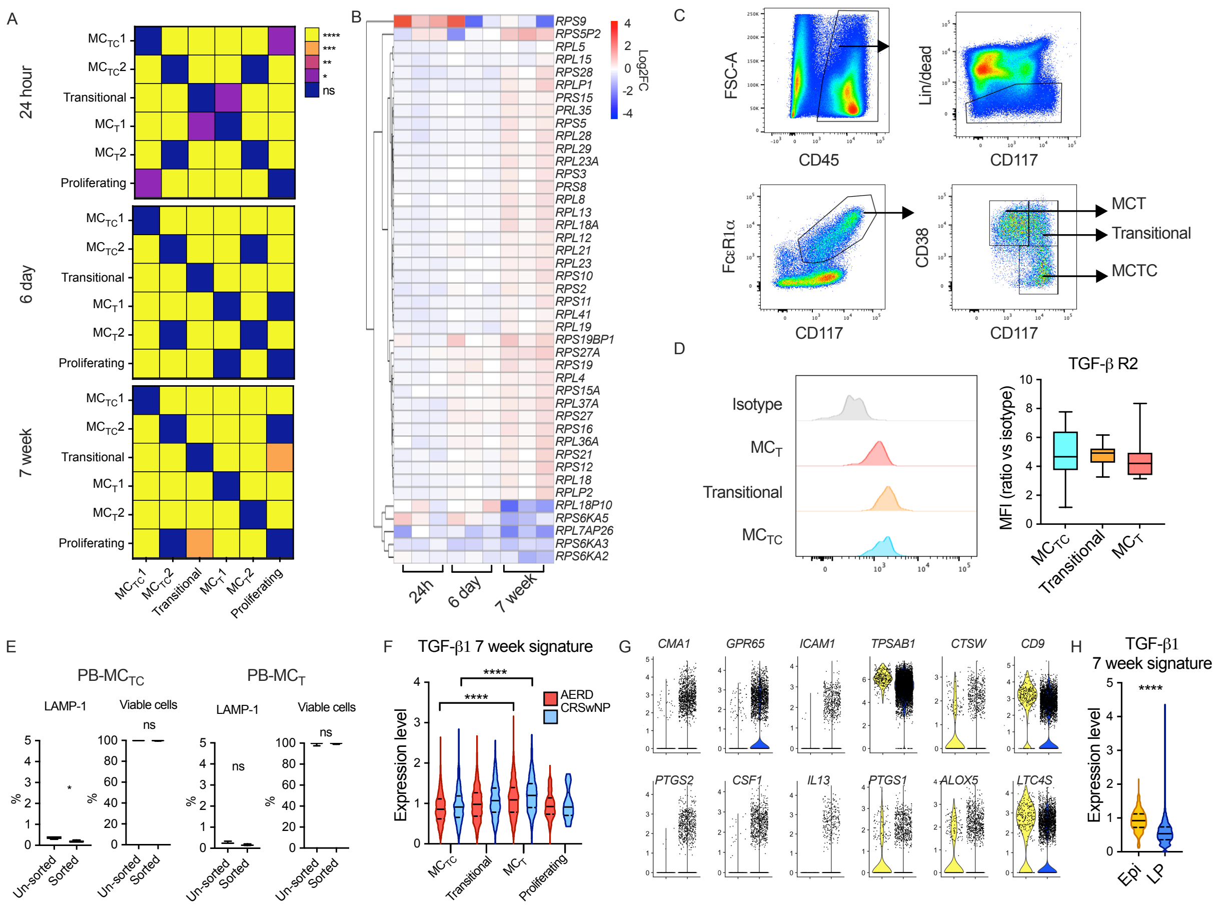

Fig. S6. Expression of TGF-β fingerprint in MCs across tissues and disease states. (**A**) Matrix showing adjusted p values for all comparisons associated with Fig. 2E (ANOVA). (**B**) Heatmap showing differentially expressed ribosomal transcripts across the indicated timepoints. Columns indicate individual donors, scale bar denotes log2FoldChange vs unstimulated controls. FDR< 0.05 (DESeq2). (**C**) Representative flow plots of gating strategy to identify MCs in nasal polyps and fractionate into MC subpopulations. (**D**) Flow cytometric assessment of cell surface expression of TGF-β R1 across the indicated populations in CRSwNP, showing representative flow vs isotype (left) and quantification of MFI ratio vs isotype (right). n= 18 individual donors (ANOVA). (**E**) Flow cytometric assessment of degranulation (measured by cell surface LAMP-1) and viability (measured using vital dye) for PB-MC_{TC} and PB-MC_T before and after flow sorting. n= 3-4 individual donors, * p< 0.05 (t-test). (**F**) Violin plots showing enrichment for seven-week TGF-β fingerprint in MC_T relative to MC_{TC} in CRSwNP and AERD. n= 6 individual donors. **** padj< 0.0001 (ANOVA). (**G**) Violin plots showing MC_T and MC_{TC}-associated transcripts differentially expressed between MCs from the epithelial vs lamina propria compartments of human colon tissue (FDR < 0.05, DESeq2) (left) and seven-week TGF-β fingerprint across tissue compartments (right). n= 30 individual donors, **** p< 0.0001 (Mann-Whitney). **(H)** Box-and-whisker plots show median, interquartile range, and minimum/maximum values observed.

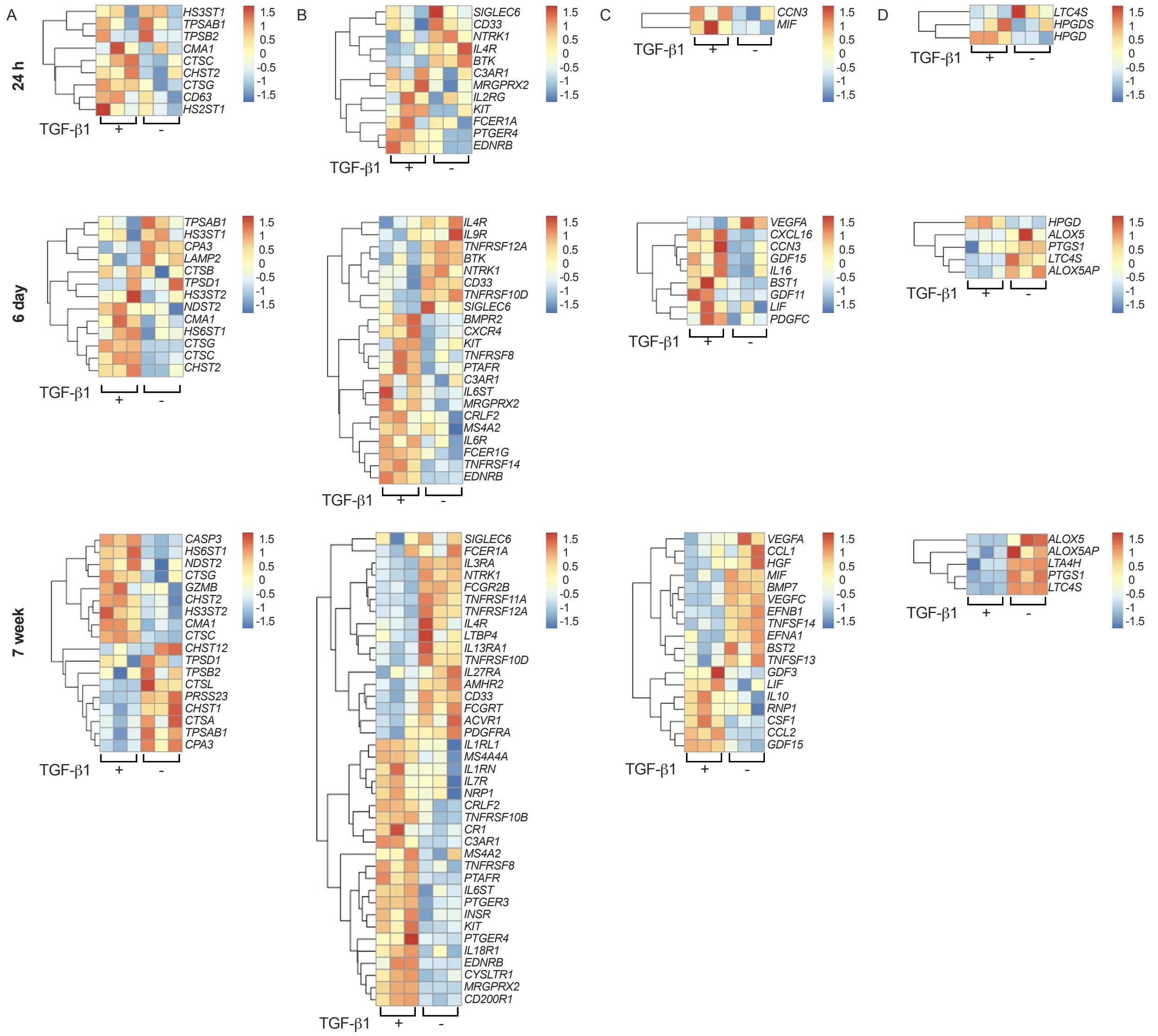

Fig. S7. Expression of genes associated with granule components, receptors, and mediators by PB-MCs. (**A-D**) Row-normalized heatmaps showing differentially expressed genes associated with (**A**) granule components, (**B**) receptors, (**C**) cytokine/chemokine/growth factors, (**D**) lipid mediator biosynthesis enzymes following treatment with TGF-β1 for 24 h (top), six day (middle), and differentiation in the presence of TGF-β1 (bottom). FDR< 0.05 (DESeq2); Data from n = 3 individual donors, with replicate numbers indicating paired samples within each experiment. Scale bars denote z score.

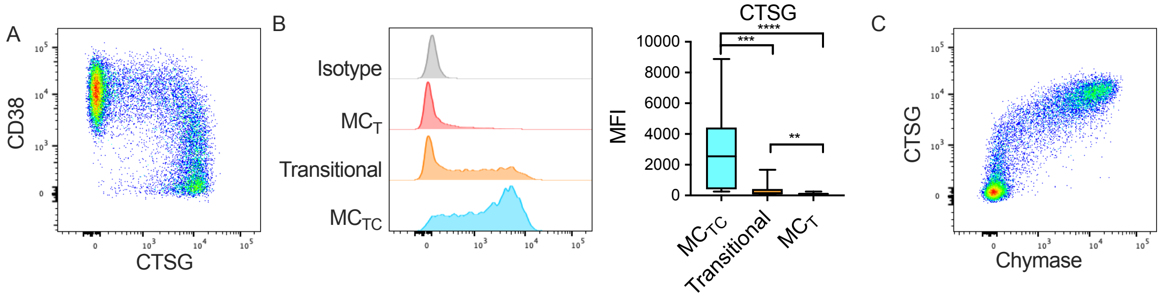

Fig. S8. Expression of CTSG by nasal polyp MC_{TC}s and MC_Ts. (**A**) Representative flow plot of CD38 vs cathepsin G, (**B**) histogram expression and quantification of cathepsin G in MC subpopulations, and (**C**) flow plot of cathepsin G vs chymase by polyp MCs. n= 12 individual donors, ** padj< 0.01, *** padj< 0.001, * padj< 0.0001 (ANOVA). Box-and-whisker plots show median, interquartile range, and minimum/maximum values observed.

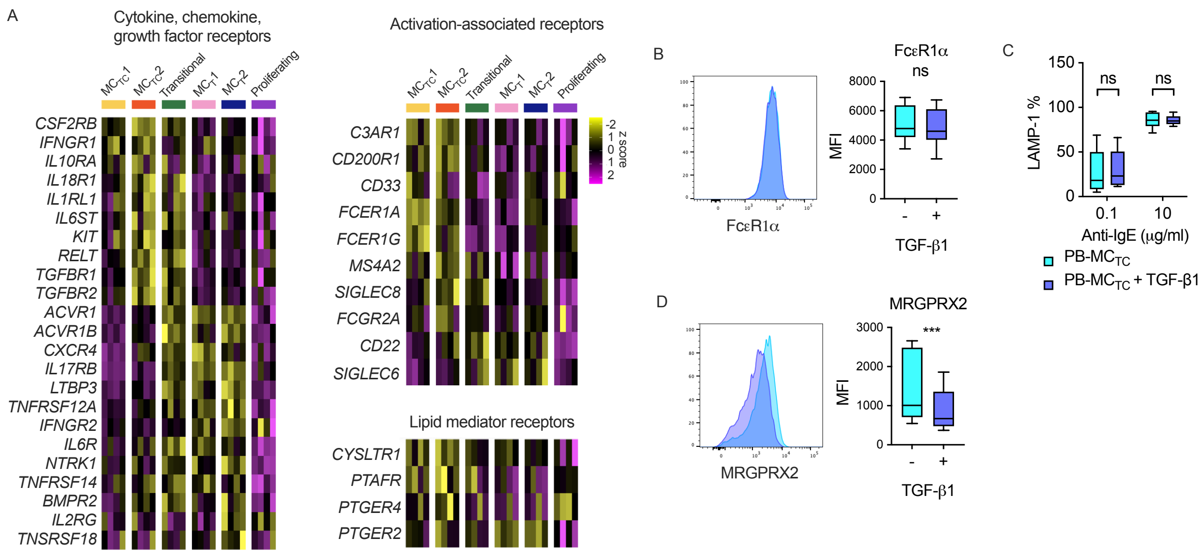

Fig. S9. Differential expression of surface receptor transcripts in nasal polyp MC subsets and effect of 24 h stimulation of PB-MC$_{TC}$s with TGF-β1. (**A**) Row-normalized heatmaps showing differentially expressed receptor-encoding transcripts in polyp MC$_{TC}$s compared with polyp MC$_T$ clusters. Columns indicate average cluster expression for each of n= 4 individuals, FDR < 0.05 (DESeq2) and Log2 Fold Change > 0.5; Scale bar indicates z score. (**B**) Representative flow plot and quantification of FcεR1α expression by PB-MC$_{TC}$s treated with (dark blue) or without (light blue) TGF-β1 for 24 h. n= 7 individual donors (t test). (**C**) Degranulation of PB-MC$_{TC}$s treated with (dark blue) or without (light blue) TGF-β1 for 24 h and activated with anti-IgE for one hour. n= 8 individual donors (ANOVA). (**D**) Representative flow plot and quantification of MRGPRX2 expression by PB-MC$_{TC}$s treated with (dark blue) or without (light blue) TGF-β1 for 24 h. n= 7 individual donors; *** p< 0.001 (ANOVA). Box-and-whisker plots show median, interquartile range, and minimum/maximum values observed.

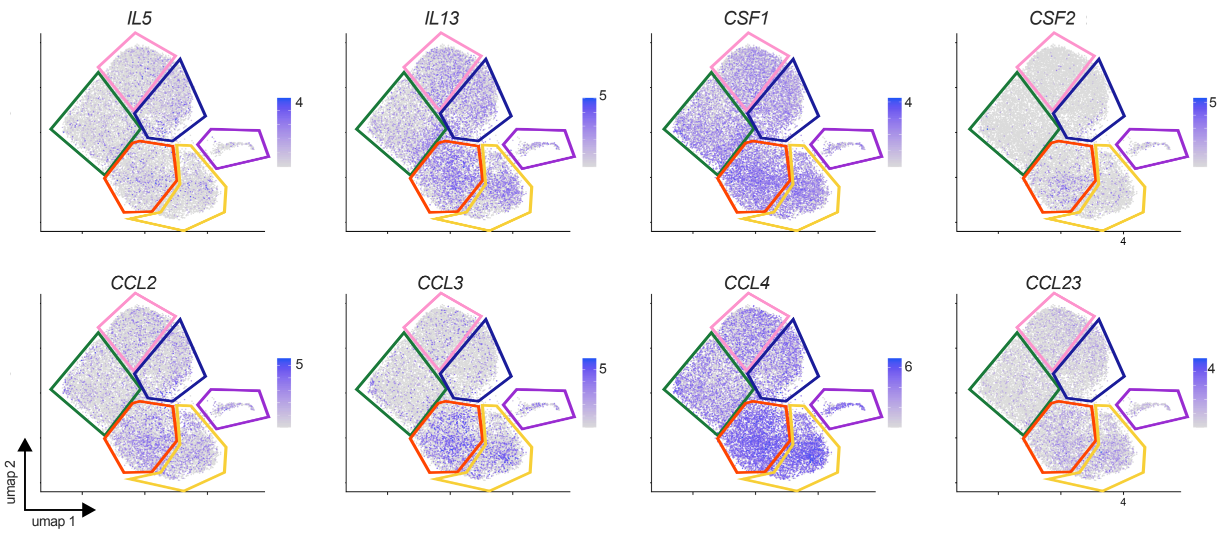

Fig. S10. Expression patterns of pro-inflammatory mediator transcripts in nasal polyp MCs. Feature Plots showing expression levels for select differentially expressed cytokines, chemokines, and growth factors across human nasal polyp MCs. Scale bar indicates normalized expression levels.

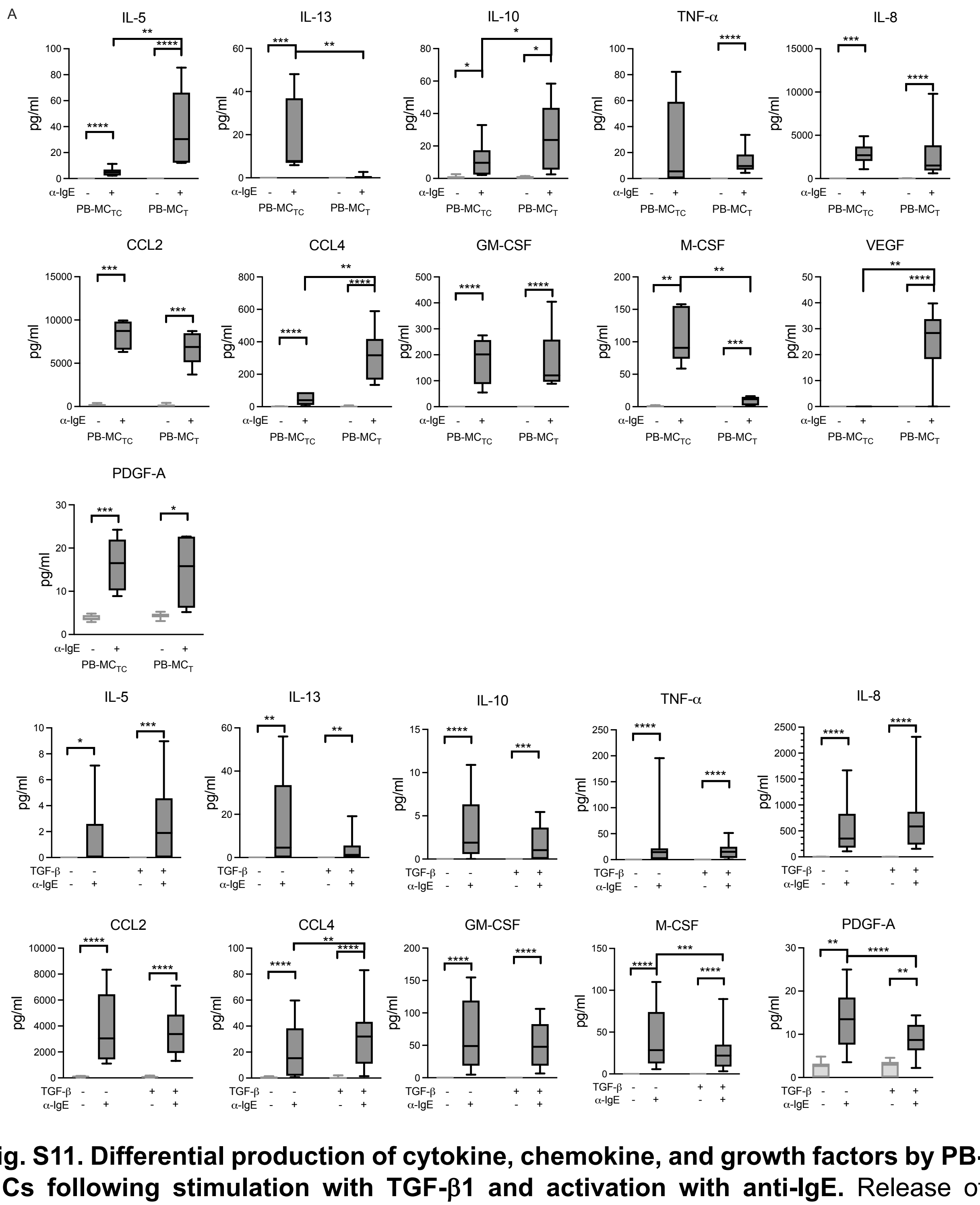

Fig. S11. Differential production of cytokine, chemokine, and growth factors by PB-MCs following stimulation with TGF-β1 and activation with anti-IgE. Release of factors produced by (**A**) PB-MC$_{TC}$s relative to PB-MC$_{T}$s and (B) PB-MC$_{TC}$s stimulated with or without TGF-β1 for six days and activated with anti-IgE for six hours. VEGF was not detected in PB-MC$_{TC}$ cultures in (**B**). n= 12-15 and 6 individual donors, respectively. * padj< 0.05, ** padj< 0.01, *** padj< 0.001, **** padj< 0.0001 (ANOVA). Box-and-whisker plots show median, interquartile range, and minimum/maximum values observed.

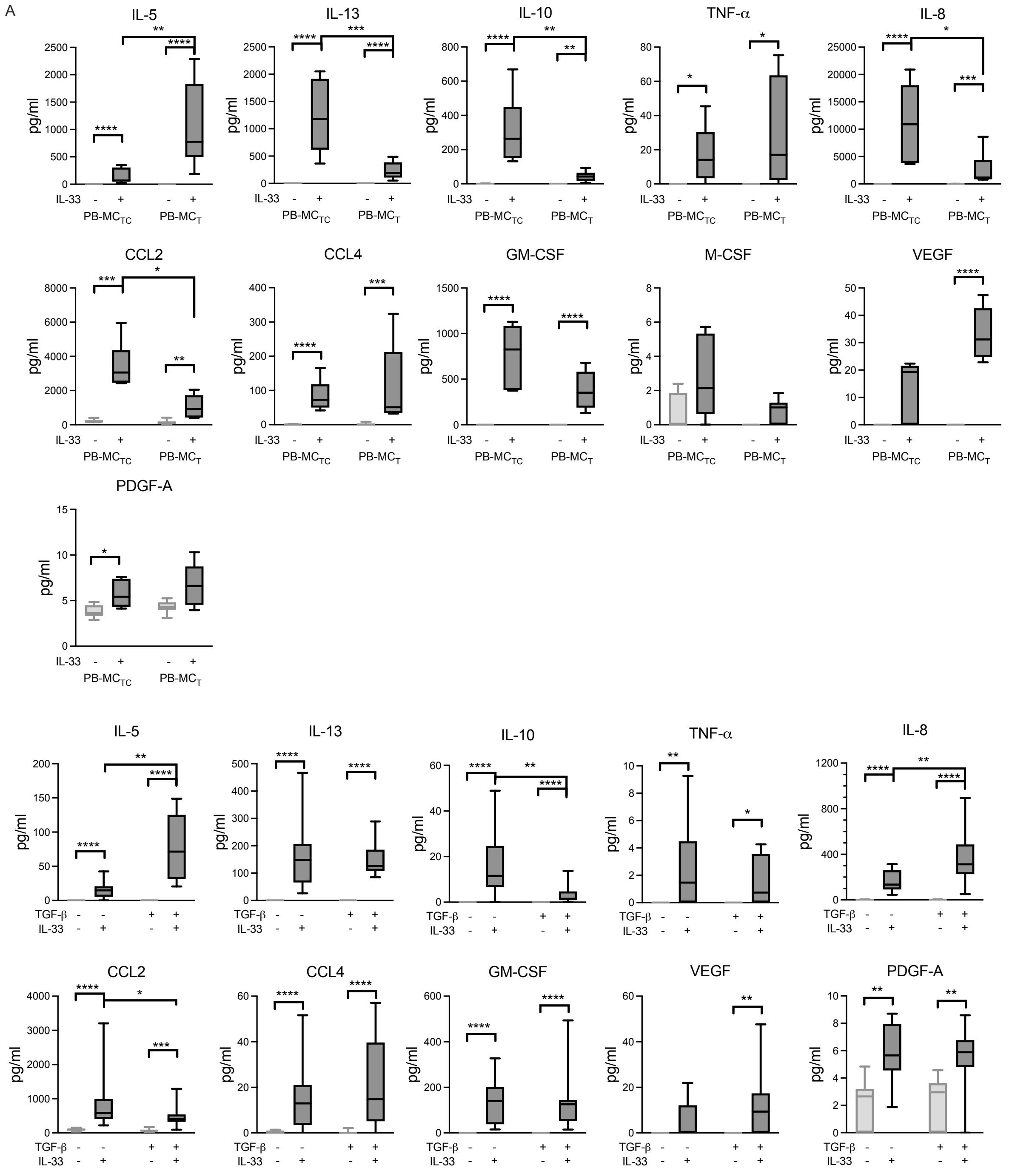

Fig. S12. Differential production of cytokine, chemokine, and growth factors by PB-MCs following stimulation with TGF-β1 and activation with IL-33. Release of factors produced by (**A**) PB-MC$_{TC}$s relative to PB-MC$_{T}$s and (**B**) PB-MC$_{TC}$s stimulated with or without TGF-β1 for six days and activated with IL-33 for six hours. M-CSF was not detected in PB-MC$_{TC}$ cultures in (B). n= 10-13 and 6 individual donors, respectively. * padj< 0.05, ** padj< 0.01, *** padj< 0.001, **** padj< 0.0001 (ANOVA). Box-and-whisker plots show median, interquartile range, and minimum/maximum values observed.

Supplementary Table 1: Differential gene expression analysis across sinonasal polyp MC clusters.

Supplementary Table 2: Pathway analysis across sinonasal polyp MC clusters and timepoint-specific TGF-β gene signatures.

Supplementary Table 3: Differential gene expression analysis between sinonasal polyp and non-polyp basal epithelial cells.

Supplementary Table 4: Differential gene expression analysis of lung basal epithelial cells from allergic asthmatic (AA) or allergic non asthmatic (ANA) patients between pre and post allergen challenge.

Supplementary Table 5: Differential gene expression analysis and gene signatures for human PB-MCs treated with or without TGF-β1 for 24 hours, six days, or differentiated in the presence of TGF-β1 for seven weeks.

Supplementary Table 6: Differential gene expression analysis of colon MCs in lamina propria versus epithelium

Supplementary Table 7: Study participant information.
